# Orthogonal Phase Transfer of Oppositely Charged Fe^II^_4_L_6_ Cages

**DOI:** 10.1002/chem.202403411

**Published:** 2024-11-07

**Authors:** Ebba S. Matic, Maylis Bernard, Alexandra J. Jernstedt, Angela B. Grommet

**Affiliations:** ^1^ Department of Chemistry and Chemical Engineering Chalmers University of Technology 412 96 Gothenburg Sweden; ^2^ Ecole Supérieure de Chimie Organique et Minérale 60200 Compiègne France

**Keywords:** Cage compounds, Ion exchange, Phase transfer, Supramolecular chemistry

## Abstract

Coordination cages and their encapsulated cargo can be manoeuvred between immiscible liquid layers in a process referred to as phase transfer. Among the stimuli reported to drive phase transfer, counterion exchange is the most widespread. This method exploits the principle that counterions contribute strongly to the solubility preferences of coordination cages, and involves exchanging hydrophilic and hydrophobic counterions. Nevertheless, phase transfer of anionic cages remains relatively unexplored, as does selective phase transfer of individual cages from mixtures. Here we compare the phase transfer behaviour of two Fe^II^_4_L_6_ cages with the same size and geometry, but with opposite charges. As such, this study presents a rare example wherein an anionic cage undergoes phase transfer upon countercation exchange. We then combine these two cages, and demonstrate that their quantitative separation can be achieved by inducing selective phase transfer of either cage. These results represent unprecedented control over the movement of coordination cages between different physical compartments and are anticipated to inform the development of next‐generation supramolecular systems.

## Graphical abstract

We report a rare example of phase transfer of an anionic coordination cage, and investigate the phase transfer behaviour of a mixture of two Fe^II^_4_L_6_ cages with the same size and geometry, but with opposite charges. These results represent unprecedented control over the movement of coordination cages between different physical compartments and are anticipated to inform the development of next‐generation supramolecular systems.

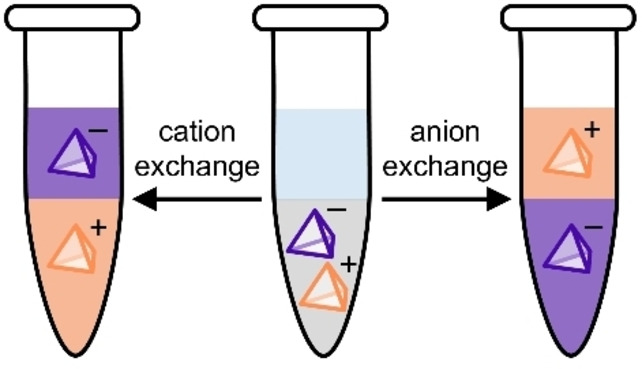

Coordination cages can move between immiscible liquid layers *via* phase transfer. As phase transfer allows encapsulated molecular cargo to be transported between distinct physical compartments,[^1^, ^2^, ^3^, ^4^, ^5^] this process has been harnessed to perform challenging chemical separations.^[6]^ The dramatic changes in solubility that drive phase transfer have been achieved by functionalizing cages with thermoresponsive moieties,^[7]^ by changing pH,^[8]^ through post‐assembly modification,^[9]^ through reversible binding of ions to the cage framework,^[10]^ and through counterion exchange.[^6^, ^8^, ^11^, ^12^, ^13^]

Among these, the counterion exchange method is the most established, and exploits the principle that counterions contribute strongly to the solubility preferences of coordination cages.[^14^, ^15^, ^16^, ^17^, ^18^, ^19^] When these cages are combined in a biphasic solvent system, their distribution between the two layers is governed by the ratio of hydrophobic and hydrophilic counterions within the system, and cages with lower charge densities transfer more readily into non‐polar solvents than cages with higher charge densities.^[12]^ When two cationic cages are combined, they have been observed to undergo phase transfer sequentially, whereby the extent to which their phase transfer profiles overlap corresponds to their difference in charge density.^[12]^ Furthermore, the counterion exchange method has been expanded beyond coordination cages to drive phase transfer of ionic organic cages^[20]^ and multi‐state dynamic coordination complexes.^[21]^

Thus far the counterion exchange driven phase transfer method has mostly been limited to cationic cages. Given the interest in using this technique for transporting and separating molecular cargo,[^1^, ^2^, ^3^, ^4^, ^5^] expanding its scope to encompass anionic cages is important, and will also unlock access to more complex supramolecular systems. Here we introduce a rare example in which countercation exchange drives phase transfer of an anionic cage. As one of the most widely used anionic cages in the field, cage **1** was selected to demonstrate the generalizability of the countercation exchange method (Figure 1),^[22]^ paving the way for the development of other phase transfer systems containing anionic cages. Furthermore, we elucidate the phase transfer behaviour of a mixed‐cage system containing anionic and cationic Fe^II^_4_L_6_ cages of the same size and geometry, where we introduce cage **2** as a cationic analogue to cage **1**. That such a system would undergo successful phase transfer was unintuitive to us, as cages bearing opposite charges could hypothetically serve as counterions for one another, thereby suppressing phase transfer. Nevertheless, when these two cages are combined, quantitative phase transfer of one species can be achieved *via* countercation or counteranion exchange, respectively, leading to complete separation of the two cages. To the best of our knowledge, this study is the first example wherein orthogonal control is exerted over the phase transfer behaviour of a mixed cage system.

**Figure 1:**
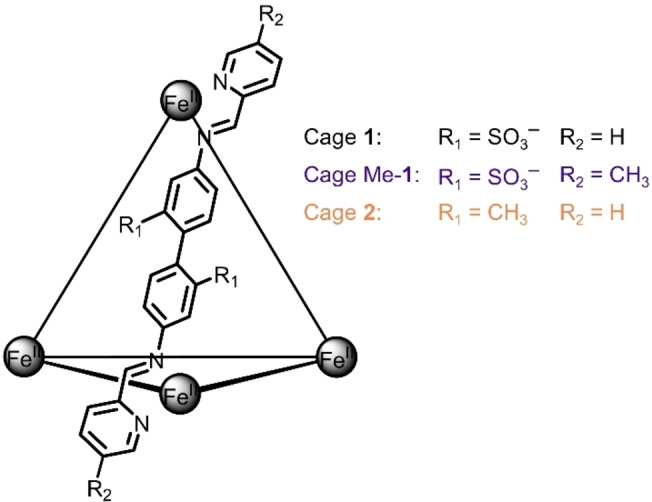
Chemical structures of cages **1**, Me‐**1**, and **2**.

Cage **2** was assembled by combining 2,2′‐dimethylbenzidine (6 equiv.), 2‐pyridine‐carboxaldehyde (12 equiv.) and iron (II) sulfate (4 equiv.) in water, and the structure was characterized using 1D and 2D NMR, ESI‐MS, and single crystal X‐ray diffraction (S2.3, S3).

The ^1^H NMR spectrum of cage **2** in D_2_O contains two distinct signals assignable to the hydrogen atoms residing on the methyl groups from the cage ligand (H_i_), at 2.40 and 2.13 ppm (Figure 2b). These two distinct chemical environments arise from methyl groups pointing roughly toward (H_i‐IN_) and away (H_i‐OUT_) from the cage cavity, resulting in a cage with both *exo*‐ and *endo*‐functionalization.[^23^, ^24^, ^25^, ^26^, ^27^] This conclusion is supported by the single crystal structure of cage **2**, wherein the methyl groups within the cage framework are oriented toward and away from the cage cavity (Figure 2a). In solution at 25 °C, the signals from H_i‐IN_ and H_i‐OUT_ are observed to undergo slow exchange on the NMR timescale, as evidenced by their crosspeaks adopting the same phase as the diagonal peaks in the ^1^H‐^1^H ROESY spectrum (Figure S8). Furthermore, the signal at 2.13 ppm is correlated to the imine signal H_e_ at 9.41 ppm, allowing us to conclusively identify this proton as H_i‐OUT_. The distance between H_i‐OUT_ and the imine proton is within the range where through‐space interactions are observable by NMR. The signals from H _i‐IN_ and H_i‐OUT_ become better resolved as temperature decreases (Figure S11), with the difference in chemical shift increasing from 0.26 ppm to 0.33 ppm upon reducing the temperature from 25 °C to 10 °C (Table S2). This increase in resolution is attributed to slower exchange rates at lower temperatures. By deconvoluting the two signals corresponding to H_i_ in the ^1^H NMR spectrum, we surmise that approximately 3 of the 12 methyl groups within each cage are oriented toward the cavity between 10 °C and 25 °C (Table S3). The external dimensions of cages **1** and **2** are very similar, with average iron‐iron distances from their single crystal structures measuring 12.847(5) Å and 12.794(5) Å respectively (Table S1). As the cavity of cage **2** is partially filled with methyl groups from the ligand, it is significantly smaller than the cavity of cage **1** (18 Å^3^ *vs*. 140 Å^3^, calculated using MoloVol,^[28]^ Figure S10).

**Figure 2:**
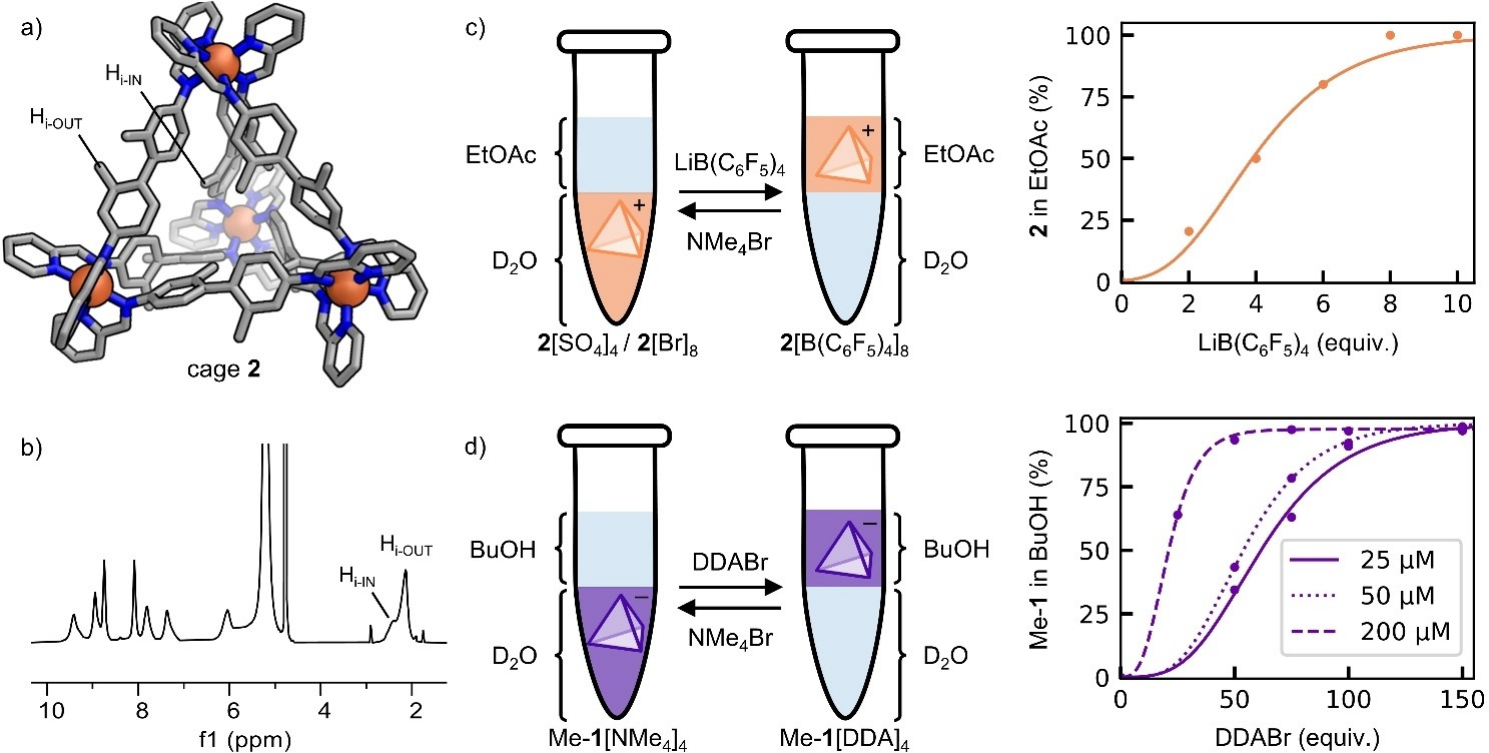
(a) Single crystal X‐ray structure of cage **2**. PF_6_^−^ counterions are omitted for clarity. (b) ^1^H NMR spectrum of cage **2**[SO_4_]_4_ in D_2_O. (c) Phase transfer of cage **2** from water into ethyl acetate, driven by counteranion exchange. (d) Phase transfer of cage Me−**1** from water into 1‐butanol, driven by countercation exchange.

Having assembled and characterized cage **2**, we began to investigate its phase transfer behaviour (Figure 2c, S7). An aqueous solution of cage **2**[SO_4_]_4_ (25 μM) was combined with an equal volume of ethyl acetate, resulting in a biphasic system where cage **2** was observed to reside exclusively in the aqueous phase; this is because cage **2**[SO_4_]_4_ is soluble in water but insoluble in ethyl acetate. Phase transfer of cage **2** from the water layer into the ethyl acetate layer could then be driven by adding an ethyl acetate solution of lithium tetrakis pentafluorophenyl borate (LiB(C_6_F_5_)_4_). Addition of this salt promotes counteranion exchange from hydrophilic SO_4_^2−^ to hydrophobic B(C_6_F_5_)_4_^−^, where cage **2**[B(C_6_F_5_)]_8_ is soluble in ethyl acetate but insoluble in water.

The minimum amount of the salt required for complete phase transfer was determined by titrating LiB(C_6_F_5_)_4_ into the biphasic system, inverting several times, allowing the layers to separate, and measuring the absorbance of cage **2** in each layer by UV‐Vis spectrometry (S7.1). The data for each layer were fitted to sigmoidal Gompertz functions (Equation 1), where *a* is the right asymptote, *b* is the steepness of the curve, *c* is the displacement of the curve along the *x*‐axis, and *d* is the left asymptote.

(1) $$y=\left(a-d\right)e^{e^{-b\left(x-c\right)}}+d$$

The curve fitted to the data corresponding to the water layer was then used to find the minimum amount of salt needed for complete transfer (S1.2). In short, the number of salt equivalents at *y*=50 % was multiplied by two, which corresponds to the point at which cage **2** is no longer detectable within the water layer. For cage **2**, 8.0 equiv. of LiB(C_6_F_5_)_4_ were required for complete transfer from water to ethyl acetate. This value is consistent with those previously reported for other cationic coordination cages.^[12]^ Furthermore, the phase transfer behaviour of cage **2** was observed to be independent of concentration between 25 μM and 5 mM, which is also consistent with previous reports.

The integrity of cage **2** in the ethyl acetate layer after phase transfer was confirmed by ^1^H NMR in non‐deuterated ethyl acetate, by locking onto the deuterium signal of D_2_O within a coaxial capillary (Figure S21). Although the aliphatic region of the spectrum is masked by signals from the solvent, there are eight signals (all with integrals of 12H) within the aromatic region of the spectrum, consistent with the eight signals from cage **2** expected in that region. Several of these signals, particularly those from the hydrogen atoms closest to the methyl group (*i. e*. H_f_, H_g_, and H_h_) are split, corresponding to different ligand conformations within the cage. Phase transfer of cage **2** from ethyl acetate back into water was accomplished by adding the salt tetramethylammonium bromide (NMe_4_Br) as a D_2_O solution dropwise, thereby promoting counteranion exchange from hydrophobic B(C_6_F_5_)_4_^−^ to hydrophilic Br^−^ (S7.3). The ^1^H NMR spectrum of cage **2** in water following transfer from ethyl acetate contains a small set of new signals from the aldehyde subcomponent, consistent with breakdown of approximately 1 % of the cage (Figure S22).

We then embarked on investigating the phase transfer behaviour of cage **1** in isolation (Figure 2d). To the best of our knowledge, there is only one other example of an anionic cage undergoing phase transfer,^[8]^ and we screened a number of different solvents and salts before coming to the conclusion that cage **1** is intrinsically too water‐soluble to readily undergo phase transfer into an organic solvent. To reduce solubility in water and improve solubility in the organic layer, we decorated the framework of cage **1** with 12 methyl groups by combining benzidine 2,2’‐disulfonic acid (6 equiv.), 5‐methyl‐2‐pyridine‐carboxaldehyde (12 equiv.), iron (II) sulfate (4 equiv.) and tetramethylammonium hydroxide pentahydrate (12 equiv.) in water to generate cage Me‐**1**[NMe_4_]_4_ (S2.2). This cage was observed to undergo phase transfer from water into 1‐butanol upon addition of the salt didodecylammonium bromide (DDABr), which drives countercation exchange from hydrophilic NMe_4_^+^ to hydrophobic DDA^+^.

To determine the minimum amount of DDABr required for complete phase transfer of cage Me−**1** from water to 1‐butanol, we performed titrations similar to the one described for cage **2** (S8.1). The integrity of cage Me−**1** in the 1‐butanol layer after phase transfer was confirmed by ^1^H NMR in deuterated 1‐butanol (S8.3). Phase transfer of cage Me−**1** from 1‐butanol back into water was accomplished by adding NMe_4_Br as a stock solution in D_2_O, thereby promoting countercation exchange from hydrophobic DDA^+^ to hydrophilic NMe^+^ (S8.3). No decomposition of cage Me−**1** was observable by ^1^H NMR.

Because DDABr is a surfactant, this set of experiments was more susceptible to emulsification than the analogous experiments for cage **2**. After inverting several times, the system was therefore centrifuged for several minutes to facilitate phase separation. We also found that these experiments were more reproducible in plastic Eppendorf or centrifuge tubes than in glass vials, likely due to interactions between DDABr and the glass surface. Notably, cage Me−**1** requires an order of magnitude more salt than cage **2** to achieve complete phase transfer at an initial concentration of 25 μM in water. Furthermore, the phase transfer behaviour of Me−**1** is concentration dependent, with higher cage concentrations requiring fewer equivalents of salt to achieve complete phase transfer. As seen in Figure 2d, complete phase transfer can be achieved using 120 equiv. of DDABr at 25 μM, 110 equiv. at 50 μM, and 42 equiv. at 200 μM.

These results suggest that the mechanisms of phase transfer driven by counteranion and countercation exchange are dramatically different. While counteranion exchange is thought to be governed predominantly by ion pairing between cage and counterion, we hypothesized that phase transfer of cage Me−**1** is also influenced by the colloidal properties of this system. To explore this effect, we performed two parallel experiments wherein DDABr was titrated into a biphasic system containing water and 1‐butanol, in the presence and absence of cage Me−**1**. Each layer was then analysed using dynamic light scattering (DLS, S8.2). Both sets of experiments contained a population below 10 nm, and a population above 1000 nm. As these sizes are both on the periphery of the range wherein DLS is considered accurate, we note only that the proportion of these two populations in 1‐butanol are dramatically different in the in the presence and absence of cage Me−**1**, with the population above 1000 nm significantly more abundant in the presence than in the absence of the cage (Figure S25).

Having elucidated the phase transfer behaviour of cages Me−**1** and **2** in isolation, we combined the two cages to investigate how each cage would influence the phase transfer behaviour of the other (Figure 3). Because the ligands within these two cages are potentially interchangeable, we first prepared a solution containing a 1 : 1 ratio of cages Me−**1** and **2** in D_2_O, and the mixture was followed by ^1^H NMR over the course of a day (Figure S30). No ligand exchange was observed on this timescale.

**Figure 3:**
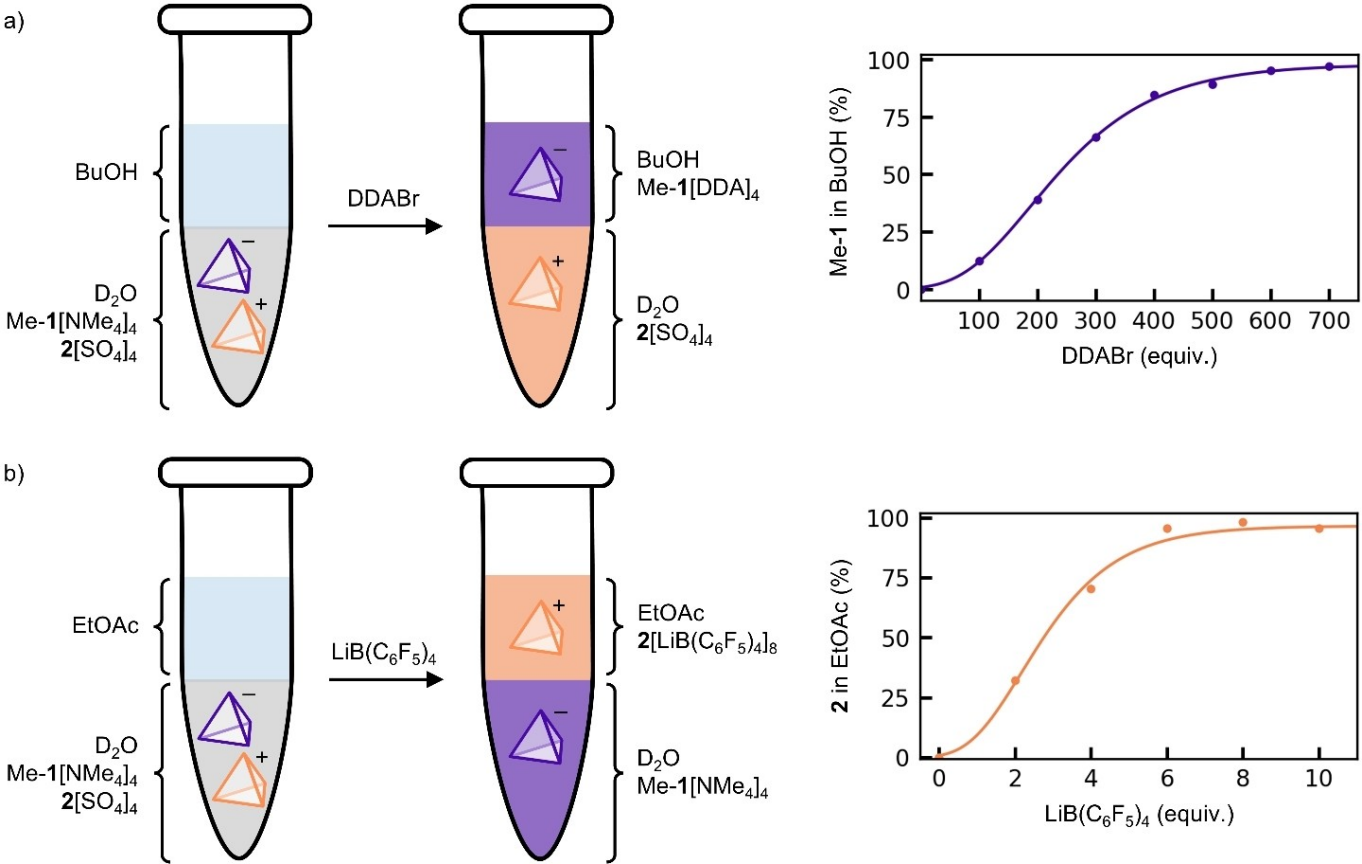
Orthogonal phase transfer of (a) cage Me−**1** or (b) cage **2** from an aqueous mixture of the two cages, to an organic layer.

We then combined an aqueous solution of the mixture (cage Me−**1**=25 μM, cage **2**=25 μM) with an immiscible organic layer, either 1‐butanol or ethyl acetate depending on which cage was intended to undergo phase transfer. Phase transfer profiles were determined by titrating salt into the biphasic system, inverting several times, centrifuging to facilitate phase separation, and measuring the absorbance of each layer by UV‐Vis spectrometry (S9.2). Addition of DDABr promoted exclusive phase transfer of cage Me−**1** from the aqueous mixture to 1‐butanol, while addition of LiB(C_6_F_5_)_4_ promoted exclusive phase transfer of cage **2** from the aqueous mixture to ethyl acetate. Both transformations resulted in quantitative separation of cage Me−**1** and cage **2**, as confirmed by ^1^H NMR (S9.3). Compared to the minimum equivalents of DDABr required for complete phase transfer of cage Me−**1** (120 equiv. at 25 μM initial concentration), significantly more salt was required to transfer cage Me−**1** away from the mixture containing cage **2** (470 equiv.). Cage **2**, however, achieved complete phase transfer with similar amounts of LiB(C_6_F_5_)_4_ in the presence (6.0 equiv.) as in the absence (8.0 equiv.) of cage Me−**1**.

In this study, we have compared the phase transfer behaviour of anionic cage Me−**1** and cationic cage **2**, driven by countercation and counteranion exchange, respectively. As opposed to the established counteranion exchange method, countercation exchange requires the addition of significantly more salt, with the minimum amount of salt required for complete phase transfer heavily dependent on cage concentration. Furthermore, we have combined cages Me−**1** and **2**, and have demonstrated orthogonal control over phase transfer of cage Me−**1** from the mixture into 1‐butanol, and over phase transfer of cage **2** from the mixture into ethyl acetate. Significantly, both transformations result in complete separation of the two cages. These findings mark a significant development in our fundamental understanding of how coordination cages undergo phase transfer, a process expected to become increasingly applicable in the field of chemical separations. Furthermore, this work is envisioned to influence the development of complex chemical systems.

## Conflict of Interests

The authors declare no conflict of interest.

## Supporting information

As a service to our authors and readers, this journal provides supporting information supplied by the authors. Such materials are peer reviewed and may be re‐organized for online delivery, but are not copy‐edited or typeset. Technical support issues arising from supporting information (other than missing files) should be addressed to the authors.

Supporting Information

## Data Availability

The data that support the findings of this study are openly available in Zenodo at https://doi.org/[10.5281/zenodo.13355534], reference number 13355533.
