# Supplementary material for: Orthogonal Phase Transfer of Oppositely Charged FeII 4L6 Cages
Source: Chemistry. 2024 Nov 7;30(71):e202403411. doi: 10.1002/chem.202403411 (PMC11653236; doi:10.1002/chem.202403411)
Supplement: Supplementary file 1 — Supporting Information [file CHEM-30-e202403411-s001.pdf]

Chemistry–A European Journal

Supporting Information

Orthogonal Phase Transfer of Oppositely Charged $Fe^{II}{}_4L_6$ Cages

Ebba S. Matic, Maylis Bernard, Alexandra J. Jernstedt, and Angela B. Grommet*

Supporting information for:

Orthogonal phase transfer of oppositely charged $Fe^{II}_4L_6$ cages

Ebba S. Matic,[a] Maylis Bernard,[b,†] Alexandra J. Jernstedt[a,†] and Angela B. Grommet*[a]

[a]Chalmers University of Technology, Department of Chemistry and Chemical Engineering, 412 96 Gothenburg, Sweden.
[b]Ecole Supérieure de Chimie Organique et Minérale, 60200 Compiègne, France.

*E-mail: angela.grommet@chalmers.se

[†]These authors contributed equally

Table of contents

S1 General ... 3
S1.1 General experimental ... 3
S1.2 Fitting of sigmoidal curves to UV-Vis data ... 3
S2 Assembly and characterization ... 4
S2.1 Cage **1** ... 4
S2.2 Cage Me-**1** ... 5
S2.3 Cage **2** ... 7
S3 X-ray crystallography and void calculations ... 11
S3.1 Structure of cage **2**$[PF_6]_8$ + $12[CH_3CN]$ ... 11
S3.2 Refinement details ... 11
S3.3 Cavity volume calculations for cages **1** and **2** ... 12
S3.4 Comparison of structures for cages **1** and **2** ... 12
S4 Variable temperature ^{1}H NMR of cage **2** ... 14
S5 UV-Vis calibration data ... 15
S5.1 Procedure ... 15
S5.2 Calibration of cage Me-**1**$[NMe_4]_4$ in water ... 16
S5.3 Calibration of cage Me-**1**$[DDA]_4$ in 1-butanol ... 17
S5.4 Calibration of cage **2**$[SO_4]_4$ in water ... 18
S5.5 Calibration of cage **2**$[B(C_6F_5)_4]_8$ in ethyl acetate ... 19
S6 Stability of cages Me-**1** and **2** ... 20
S6.1 Procedure and summary of results ... 20
S6.2 Stability of cage Me-**1**$[NMe_4]_4$ in water ... 20
S6.3 Stability of cage Me-**1**$[DDA]_4$ in 1-butanol ... 21
S6.4 Stability of cage **2**$[SO_4]_4$ in water ... 22
S6.5 Stability of cage **2**$[B(C_6F_5)_4]_8$ in ethyl acetate ... 23
S7 Phase transfer of cage **2** ... 24
S7.1 Anion titration followed by UV-Vis spectroscopy ... 24
S7.2 ^{1}H NMR of cage **2** in ethyl acetate ... 27
S7.3 ^{1}H NMR of cage **2** in water, following transfer from ethyl acetate ... 28
S8 Phase transfer of cage Me-**1** ... 29
S8.1 Cation titration followed by UV-Vis spectroscopy ... 29
S8.1.1 Procedure and summary of results ... 29
S8.1.2 Phase transfer of cage Me-**1**, 25 μM ... 30
S8.1.3 Phase transfer of cage Me-**1**, 50 μM ... 32
S8.1.4 Phase transfer of cage Me-**1**, 200 μM ... 34
S8.2 Cation titration followed by DLS ... 36
S8.2.1 Procedure and summary of results ... 36
S8.2.2 Size of peaks 1 and 2 in the presence and absence of cage Me-**1** in 1-butanol ... 37
S8.2.3 Integrals of peaks 1 and 2 in the presence and absence of cage Me-**1** in 1-butanol ... 38
S8.2.4 Representative DLS spectra ... 39
S8.3 ^{1}H NMR of cage Me-**1** in 1-butanol ... 40
S9 Orthogonal phase transfer ... 43
S9.1 ^{1}H NMR spectra of cages Me-**1** and **2** over time ... 43
S9.2 Orthogonal phase transfer followed by UV-Vis spectrometry ... 44
S9.2.1 Procedure and summary of the data ... 44
S9.2.2 Selective transfer of cage Me-**1** from the mixture ... 45
S9.2.3 Selective transfer of cage **2** from the mixture ... 47
S9.3 Orthogonal phase transfer followed by NMR spectroscopy ... 49
S10 Supporting references ... 52

S1 General

S1.1 General experimental

All solvents and reagents were obtained from commercial sources and used as supplied unless otherwise noted. The water and D_2O used in cage assembly was degassed using three evacuation/N_2 fill cycles before use. ^1H and $^{13}C\{^1H\}$ NMR spectra were recorded on a 600 MHz Bruker Avance NEO spectrometer or on an 800 MHz Bruker Avance III HD spectrometer with an Oxford magnet. ^1H NMR shifts (δ_H) are expressed in parts per million (ppm) and reported relative to an external standard, either *tert*-butanol (*t*-BuOH) (δ_H = 1.24 ppm) or dextran sulphate sodium salt (DSS) (δ_H = 0.00 ppm) in a coaxial D_2O capillary. $^{13}C\{^1H\}$ chemical shifts (δ_C) are expressed in ppm and reported relative to the external standard *t*-BuOH (δ_C = 70.36 ppm and 30.29 ppm) in a coaxial D_2O capillary. Abbreviations used when describing NMR spectra are as follows: s, singlet; bs, broad singlet; d, doublet; m, multiplet. UV-Vis spectra were collected on a Varian Cary 50 UV-Vis spectrophotometer. All measurements were performed using Brand plastic semi-micro UV cuvettes, with a 1 cm path length. These cuvettes are resistant to organic solvents, including 1-butanol and ethyl acetate. Dynamic light scattering (DLS) was performed on a Malvern Zetasizer Ultra. All NMR, UV-Vis, and DLS measurements were carried out at 298 K. High resolution electrospray ionization mass spectrometry (ESI-MS) was performed on a Waters Select series cyclic IMS, run as QTOF. The sample was direct infused with an external infusion pump at 20 µl/min. The data was corrected using leucine-enkephaline reference solution and AFAMM data treatment.

S1.2 Fitting of sigmoidal curves to UV-Vis data

Sigmoidal Gompertz functions (Equation S1) were fitted to the UV-Vis data for each layer. The Gompertz function is a sigmoidal function where the right-side asymptote (*a*) is approached more gradually than the left side asymptote (*d*). *b* is the growth rate and *c* is the displacement of the curve along the x-axis.

$$y = (a - d)e^{e^{-b(x-c)}} + d \qquad \text{Equation S1}$$

The Gompertz functions are fitted to the data using the curveFitter tool in MatLab. The terms *a* and *d* represent the maximum and minimum amount of cage in the layer, depending on whether the cage is transferring into or out of the layer. As the maximum amount of cage in a layer is 100%, the upper limit of the upper asymptote was set to 100. As the minimum amount is 0%, the lower limit for the lower asymptote was set to 0. When the data indicates complete transfer, the upper asymptote was set to 100. When the data indicates that transfer is incomplete, the upper asymptote was left undefined.

The minimum number of equivalents (equiv.) needed for complete transfer was determined by multiplying the value at y = 50% by two.

S2 Assembly and characterization

S2.1 Cage 1

Cage **1** was assembled following the procedure described previously.[29] 2-Pyridine-carboxaldehyde (0.349 mL, 5.59 mmol), benzidine 2,2'-disulfonic acid (15 wt% water, 0.710 g, 1.79 mmol), iron (II) sulphate heptahydrate (0.332 g, 1.92 mol) and tetramethylammonium hydroxide pentahydrate (0.645 g, 5.59 mmol) were combined in a round bottom flask containing 25 mL degassed MiliQ water. The dark purple solution was stirred under nitrogen at 50 °C for 20 h. The solution was removed from the heat and allowed to cool. The product was then isolated as a dark purple solid by precipitation with dry acetone.

^{1}H NMR (600 MHz, D_2O, referenced to *t*-BuOH): δ_H = 9.37 (apparent s, 12H, H_e), 8.74 (apparent s, 12H, H_d), 8.44 (apparent s, 12H, H_c), 7.80 (bs, 12H, H_b), 7.56 (apparent s, 12H, H_a), 7.17 (bs, 12H, H_g), 6.48 (apparent s, 12H, H_h), 5.87 (apparent s, 12H, H_f), 3.22 (s, 48H, $[NMe_4]^+$).

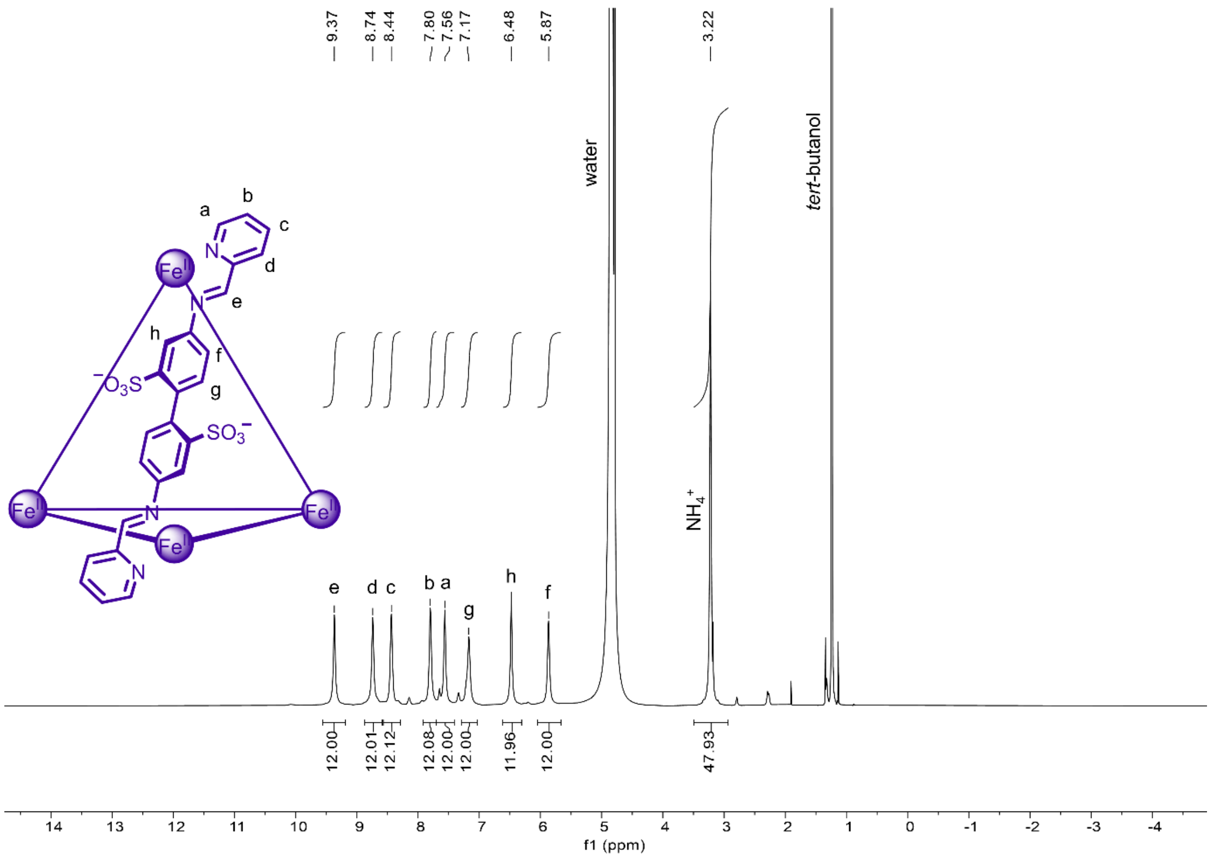

Figure S1. ^{1}H NMR (600 MHz, D_2O, 298 K, referenced to *t*-BuOH) of cage **1**$[NMe_4]_4$.

S2.2 Cage Me-1

5-Methylpyridine-2-carboxaldehyde (72.7 mg, 6.00 mmol), benzidine 2,2'-disulfonic acid (15 wt% water, 134 mg, 3.90 mmol), iron (II) sulphate heptahydrate (55.6 mg, 2.00 mmol) and tetramethylammonium hydroxide heptahydrate (109 mg, 6.00 mmol) were combined in a vial containing 10 mL degassed MiliQ water. The dark purple solution was stirred under nitrogen at 50 °C for 20 h. The solution was removed from the heat and allowed to cool before the product was isolated by precipitation with cold acetone. The solid was washed with acetone to remove excess tetramethyl ammonium.

^{1}H NMR (600 MHz, D_2O, referenced to *t*-BuOH): δ_H = 9.28 (s, 12H, H_e), 8.59 (d, 12H, *J* = 7.9 Hz, H_d), 8.22 (d, 12H, *J* = 7.9 Hz, H_c), 7.29 (s, 12H, H_a), 7.12 (apparent s, 12H, H_g), 6.43 (s, 12H, H_h), 5.80 (apparent s, 12H, H_f), 3.20 (s, 48H, NMe_4^+) 2.36 (s, 36H, H_b).

^{13}C{^{1}H} NMR (151 MHz, D_2O, referenced to *t*-BuOH): δ_C = 176.28, 156.98, 156.32, 151.24, 143.90, 142.94, 140.92, 136.82, 132.92, 132.28, 122.85, 121.84, 56.15, 19.50.

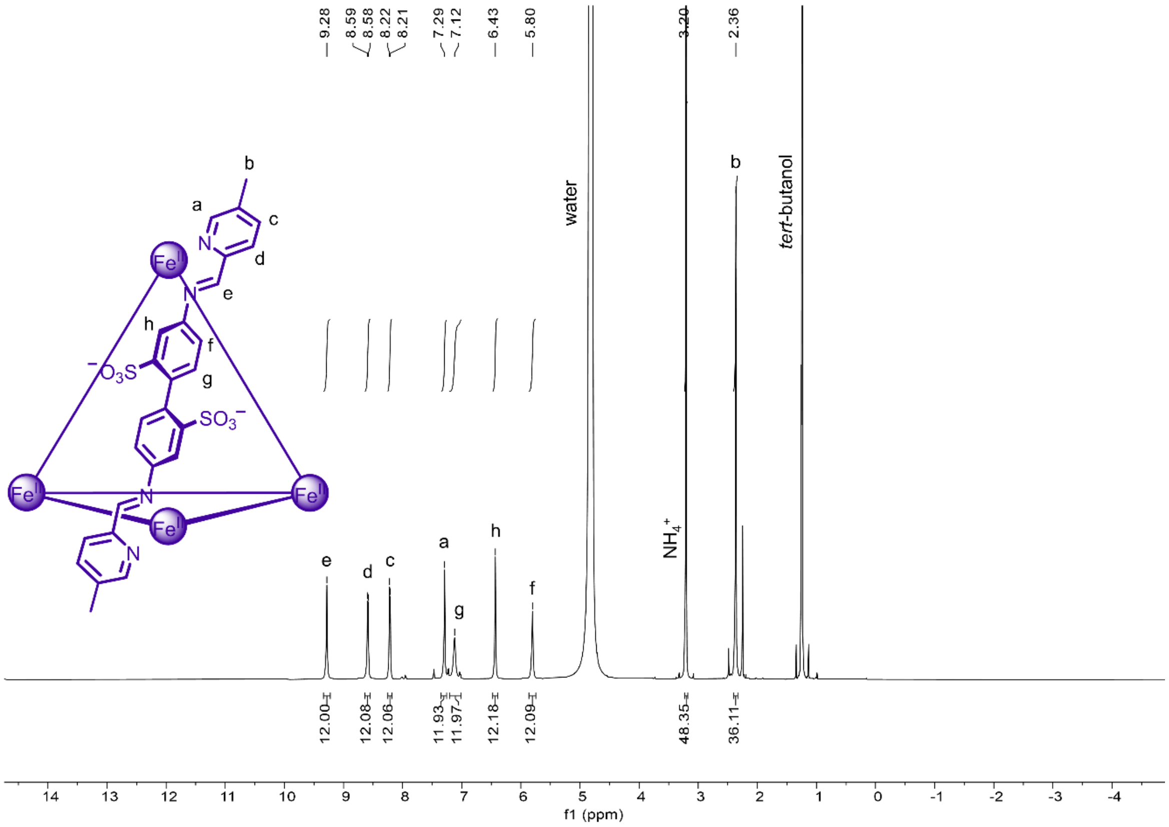

Figure S2. ^{1}H NMR (600 MHz, D_2O, 298K, referenced to *t*-BuOH) of cage Me-**1**$[NMe_4]_4$.

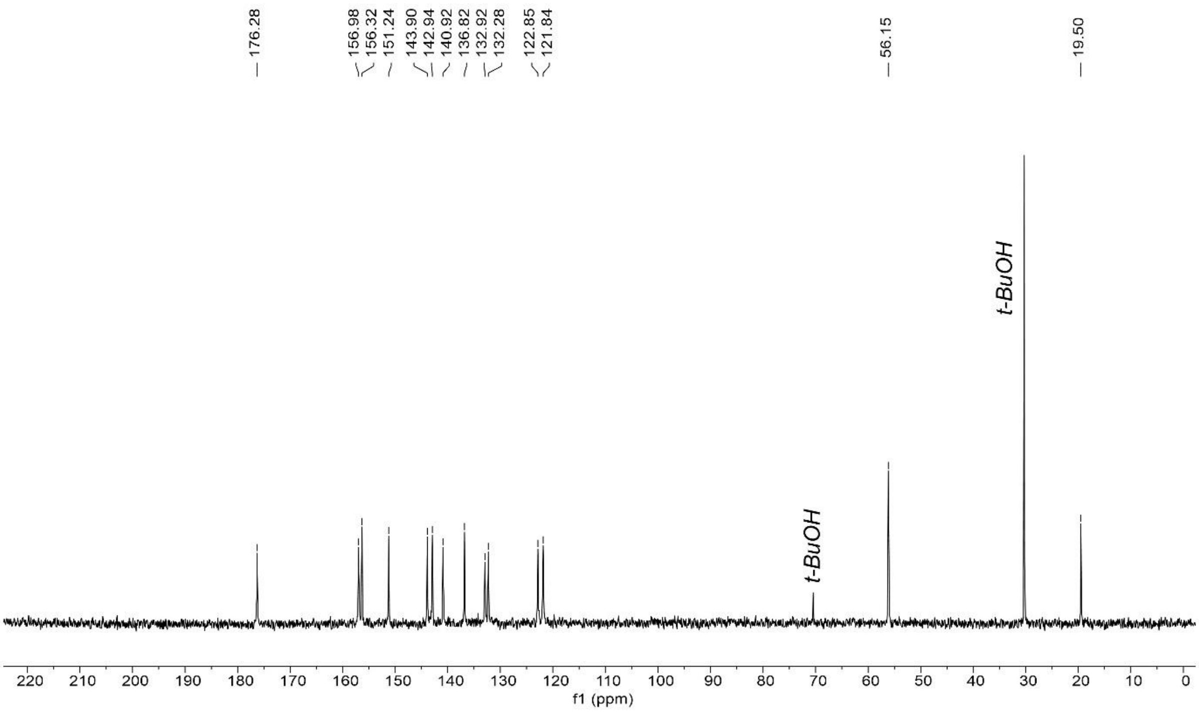

Figure S3. $^{13}C\{^{1}H\}$ NMR (151 MHz, D_2O, 298K, referenced to *t*-BuOH) of cage Me-**1**$[NMe_4]_4$.

S2.3 Cage 2

2,2'-Dimethylbenzidine (63.7 mg, 300 µmol), iron (II) sulphate heptahydrate (55.6 mg, 200 µmol) and 2-pyridine-carboxaldehyde (57.1 µL, 600 µmol) were added to 5 mL of D_2O water in a round bottom flask and stirred at 50 °C for 20 h. For phase transfer experiments, the cage solution was used without further purification. For characterization by ESI-MS and to grow single crystals for X-ray diffraction, the cage was precipitated out of aqueous solution using KPF_6, then redissolved in acetonitrile.

^1H NMR (800 MHz, D_2O, referenced to DSS): δ_H = 9.41 (s, 12H, H_e), 8.94 (apparent s, 12H, H_d), 8.74 (apparent s, 12H, H_c), 8.08 (apparent s, 12H, H_b), 7.81 (apparent s, 12H, H_a), 7.37 (apparent s, 12H, H_g), 6.04 (bs, 12H, H_f), 5.71 (bs, 12H, H_h), 2.40 and 2.13 (bs and bs, total 36H, $H_{i\text{-IN}}$ and $H_{i\text{-OUT}}$ respectively).

^1H NMR (600 MHz, CD_3CN, referenced to acetonitrile): δ_H = 9.10 (s, 12H, H_e), 8.60 (apparent s, 12H, H_d), 8.38 (apparent s, 12H, H_c), 7.82 (apparent s, 12H, H_b), 7.54 (apparent s, 12H, H_a), 6.95 (apparent s, 12H, H_g), 6.04 (apparent s, 12H, H_f), 5.71 (bs, H_h), 2.16 and 1.83 (bs and bs, 36H, H_i).

$^{13}C\{^1H\}$ NMR (151 MHz, D_2O, referenced to *t*-BuOH): δ_C =175.81, 159.43, 156.78, 151.37, 141.41, 140.94, 140.51, 138.73, 131.85, 130.55, 124.22, 119.20, 19.77.

ESI-MS: **2**$[PF_6]_6^{2+}$ 1717.3158 (calc. 1717.3221); **2**$[PF_6]_5^{3+}$ 1096.5558 (calc. 1096,5583); **2**$[PF_6]_4^{4+}$ 786.1758 (calc. 786.179); **2**$[PF_6]_1^{7+}$ 387.1158 (calc. 387.1178).

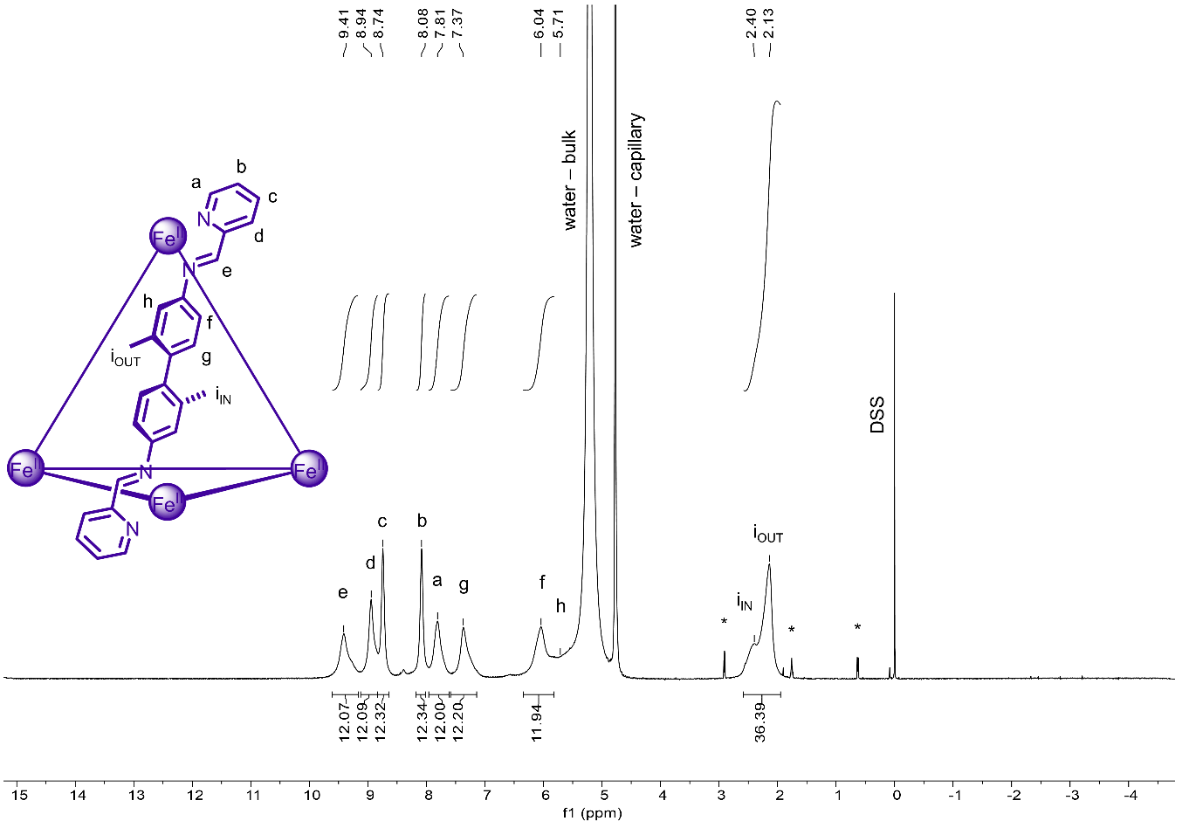

Figure S4. ^1H NMR (800 MHz, D_2O, 298 K, referenced to DSS) of cage **2**$[SO_4]_4$. Signals marked with * are also from DSS. As signal from H_h is extremely broad and overlaps with the signal from water, the integral is not reported. Assignments are supported by the ^1H-^1H COSY spectrum in Figure S7.

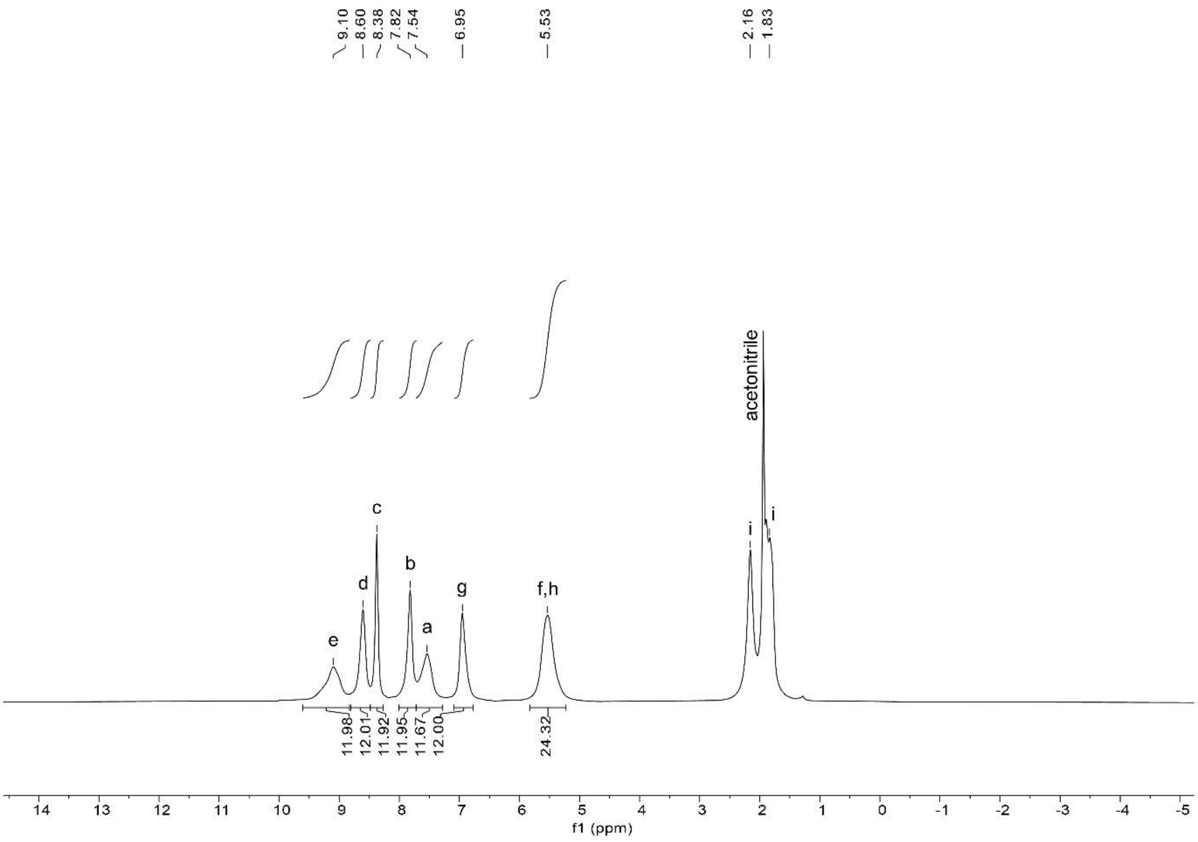

Figure S5. ^{1}H NMR (600 MHz, D_2O, 298 K, referenced to acetonitrile) of cage **2**$[PF_6]_8$. As the residual acetonitrile signal overlaps with the signal for H_i, the integral for this signal is not reported.

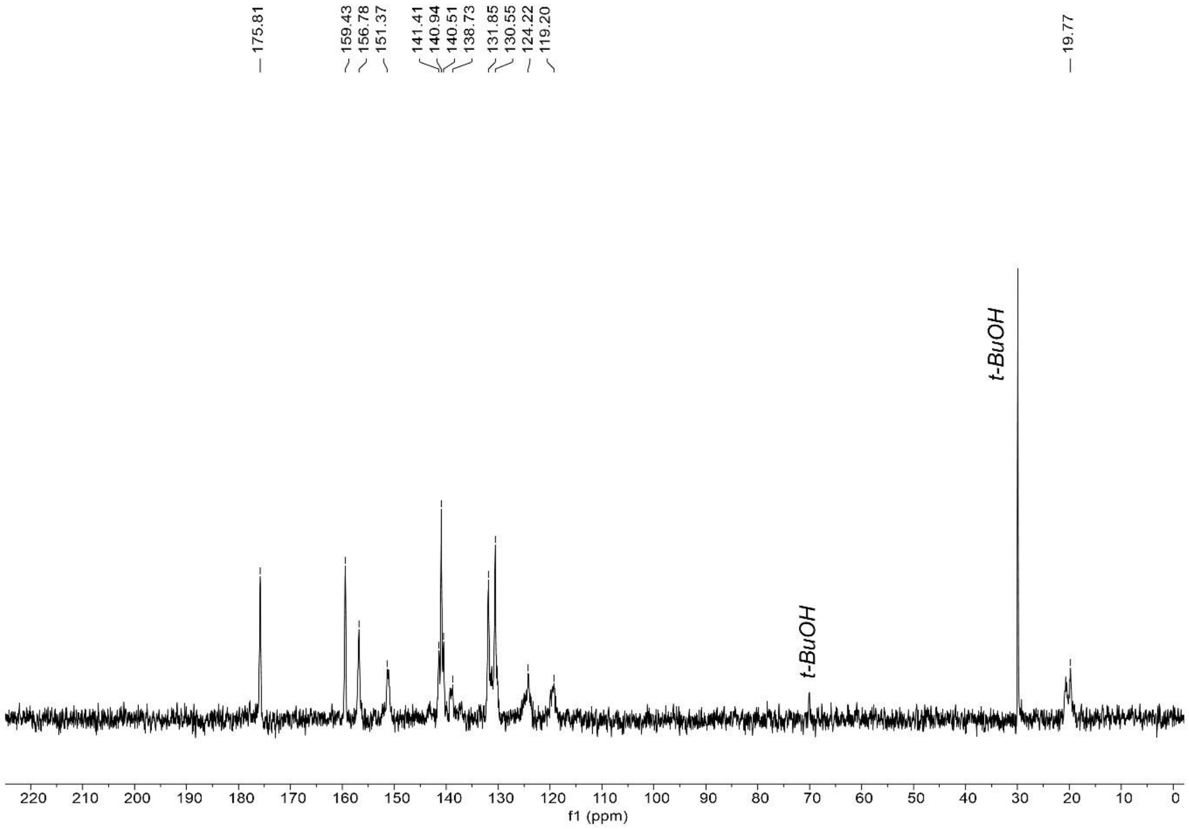

Figure S6. $^{13}C\{^{1}H\}$ NMR (151 MHz, D_2O, 298K, referenced to *t*-BuOH) of cage **2**$[SO_4]_4$.

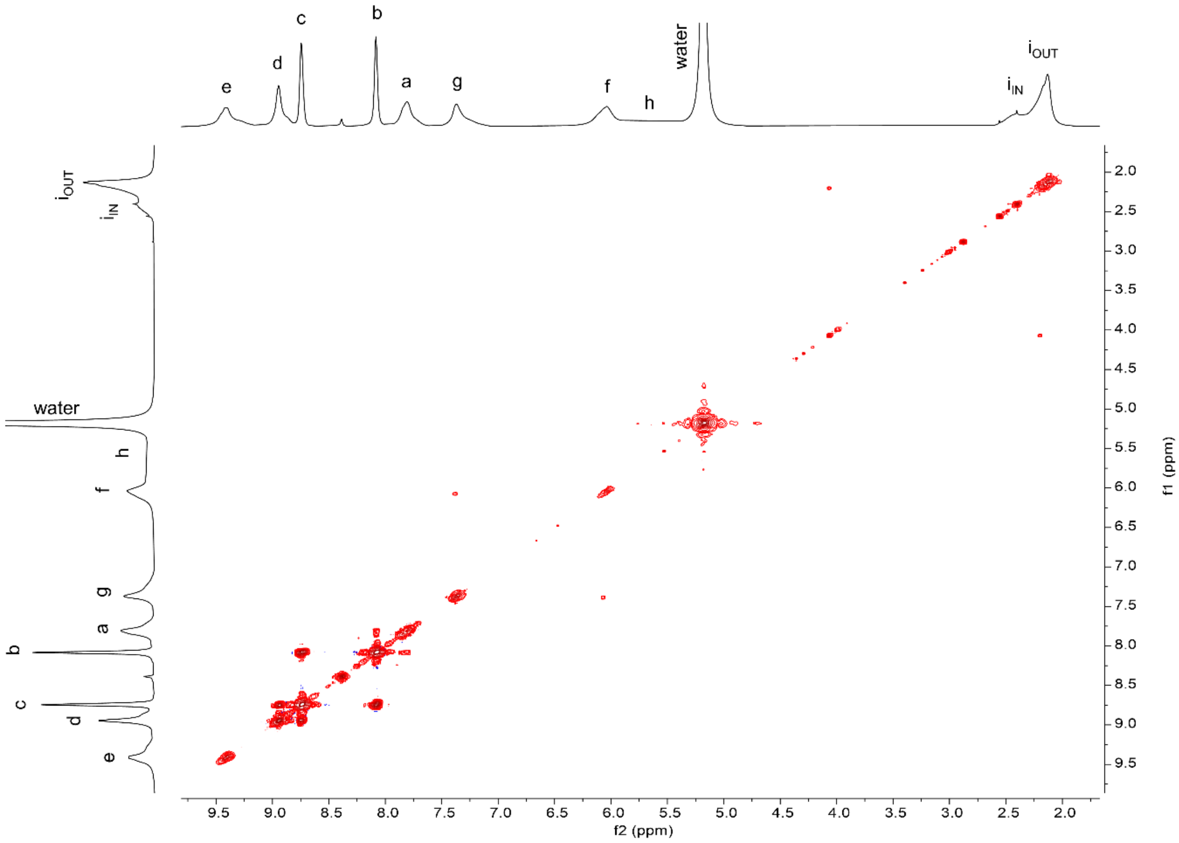

Figure S7. ^{1}H-^{1}H COSY (800 MHz, D_2O, 298 K, referenced to DSS) of cage **2**$[SO_4]_4$.

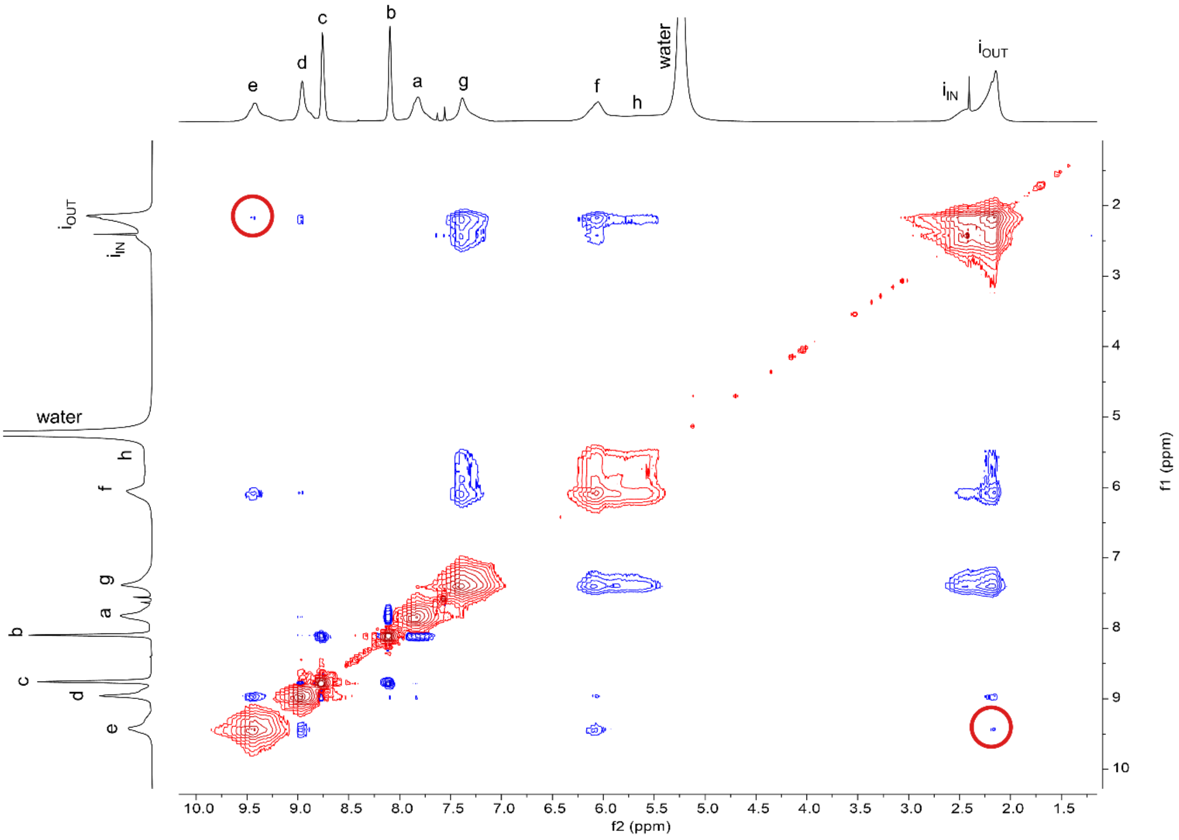

Figure S8. ^{1}H-^{1}H ROESY (800 MHz, D_2O, 298 K, referenced to DSS) of cage **2**$[SO_4]_4$.

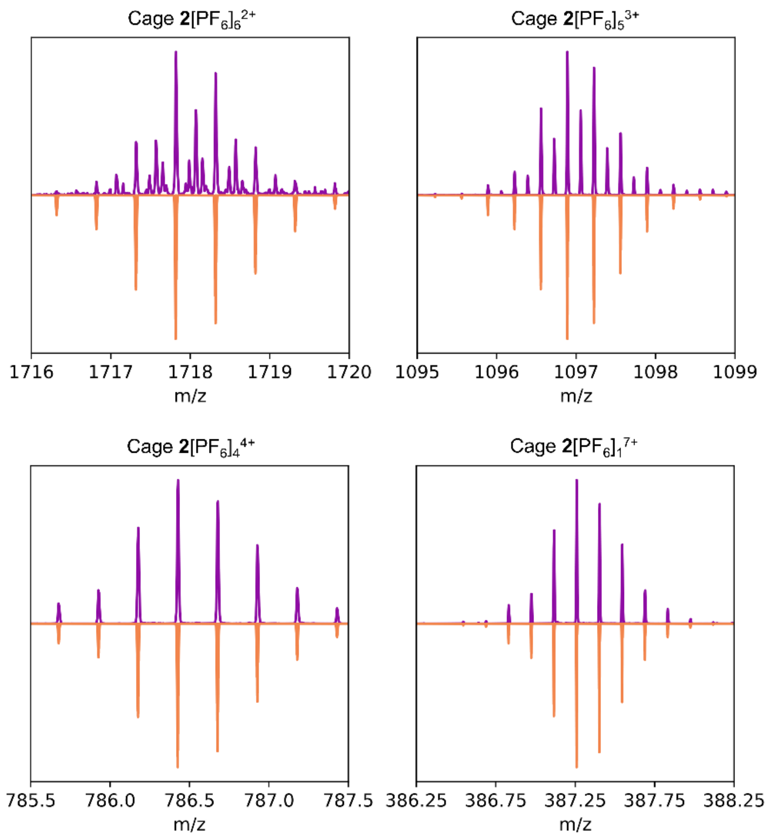

Figure S9. ESI-MS of cage **2**[PF_6]$_8$, showing experimental (top, purple) *vs.* calculated (bottom, orange) isotope distributions values for the 2+, 3+, 4+, and 7+ species.

S3 X-ray crystallography and void calculations

S3.1 Structure of cage 2[PF_6]$_8$ + 12[CH_3CN]

Empirical formula	$C_{182}H_{171}F_{48}Fe_4N_{37}P_8$
Formula weight	4259.71
Temperature/K	160.15
Crystal system	monoclinic
Space group	$P2_1/c$
a/Å	31.5899(11)
b/Å	19.3496(4)
c/Å	35.2702(15)
α/°	90
β/°	116.429(5)
γ/°	90
Volume/Å^3	19305.8(14)
Z	4
ρ_{calc}g/cm^3	1.296
μ/mm^{-1}	3.845
F(000)	7653.0
Crystal size/mm^3	0.29 × 0.14 × 0.04
Radiation	CuKα (λ = 1.54184)
2Θ range for data collection/°	5.05 to 136.5
Index ranges	$-38 \leq h \leq 38$, $-23 \leq k \leq 12$, $-42 \leq l \leq 42$
Reflections collected	173993
Independent reflections	35285 [R_{int} = 0.1386, R_{sigma} = 0.0767]
Data/restraints/parameters	35285/1669/2235
Goodness-of-fit on F^2	1.491
Final R indexes [I>=2σ (I)]	R_1 = 0.1464, wR_2 = 0.3985
Final R indexes [all data]	R_1 = 0.2062, wR_2 = 0.4369
Largest diff. peak/hole / e Å^{-3}	1.94/-1.16

S3.2 Refinement details

Single crystals were grown by slow vapor diffusion of ethyl acetate into an acetonitrile solution of **2**[PF_6]$_8$ at 4 °C. This gave dark purple plate shaped crystals. The crystals lost solvent immediately upon removal from the mother liquor, and rapid handling before the crystal was placed in the cryostream was required to collect data.

Data were collected using Cu Kα radiation (λ = 1.54184) on a XtaLAB Synergy, Dualflex, Pilatus 200K diffractometer at 160.15 K. Data integration and reduction were performed using CrysAlisPro 1.171.43.117a (Rigaku Oxford Diffraction, 2024). An analytical absorption correction was applied. The diffraction intensity was low, and few reflections were observed at high angles. However, data quality was sufficient to determine connectivity within the cage.

The structure was solved using Olex2[30], with the olex2.solve structure solution program[30] using Charge Flipping and subsequently refined with the SHELXL package[31] using least squares minimisation. Non-hydrogen atoms were refined aniisotropically. Hydrogen atoms were refined using a riding model.

Two of methyl groups show significant signs of disorder. These were modelled as occupying two positions on either side of their phenyl rings, both positions with an occupancy of approximately 0.5. The occupancy was freely refined. A thermal parameter restraint (RIGU) was applied to the phenyl rings connected to these methyl groups. Several of the other methyl groups also show signs of disorder, however, due to the limited resolution of the data this disorder could not be modelled. This disorder corresponds to twisting of the phenyl groups in the amine component in the crystal.

The hexafluorophosphate counterions were modelled as rigid groups (DFIX, RIGU applied)[32] with idealized structures disordered over two positions with the occupancy freely refined, or as not disordered. There is a significant amount of disordered solvent that could not be modelled, only one acetonitrile molecule could be found. The BYPASS function[33] in OLEX2 was therefore used to remove the contribution of electron density from unresolved solvent molecules. A solvent mask was calculated and 1092 electrons were found in a volume of 4288Å^3 in 4 voids per unit cell. This is consistent with the presence of 12 acetonitrile molecules per formula unit which account for 1056 electrons per unit cell.

CheckCIF gives 5 B level alerts which result from the low resolution of the data, and the disorder in the ligands. The data was checked for possible twinning, which could account for some of the alerts. There is no twinning.

Crystallographic data have been deposited with the CCDC (2378426).

S3.3 Cavity volume calculations for cages 1 and 2

The voids for cage **1**[29] and cage **2** were calculated using the program MoloVol[329 using a two-probe model. The small probe is 1.2 Å, the large is 3.0 Å, the grid resolution is 0.1 and the optimization depth is 4.

Elemental radii:

C 1.770000
F 1.460000
Fe 2.440000
H 1.200000
N 1.660000
P 1.900000

S3.4 Comparison of structures for cages 1 and 2

Table S1. Comparison of cages **1** and **2.** Cavity volumes are calculated as described in section S3.3.

	Cage **1**	Cage **2**
Fe1–Fe2 (Å)	12.892(2)	12.800(2)
Fe1–Fe3 (Å)	12.888(3)	12.836(2)
Fe1–Fe4 (Å)	12.899(2)	12.836(2)
Fe2–Fe3 (Å)	12.830(2)	12.710(2)
Fe2–Fe4 (Å)	12.889(2)	12.772(2)
Fe3–Fe4 (Å)	12.846(2)	12.812(2)
Average Fe–Fe (Å)	12.847(5)	12.794(5)
Cavity volume (Å^3)	141.12	17.51

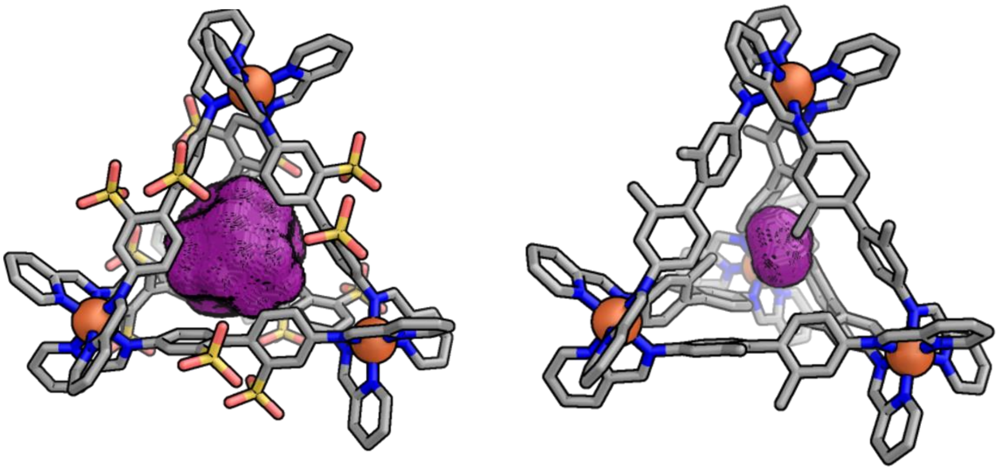

1
141 Å^3

2
17.5 Å^3

Figure S10. Rendered images of cages **1** and **2** with cavities depicted as purple surfaces.

S4 Variable temperature ^{1}H NMR of cage 2

The following variable temperature (VT) ^{1}H NMR data of cage **2**[SO_4]$_4$ in D_2O was collected on an 800 MHz Bruker Avance III HD spectrometer with an Oxford magnet. The sample was equilibrated for 10 minutes at each temperature.

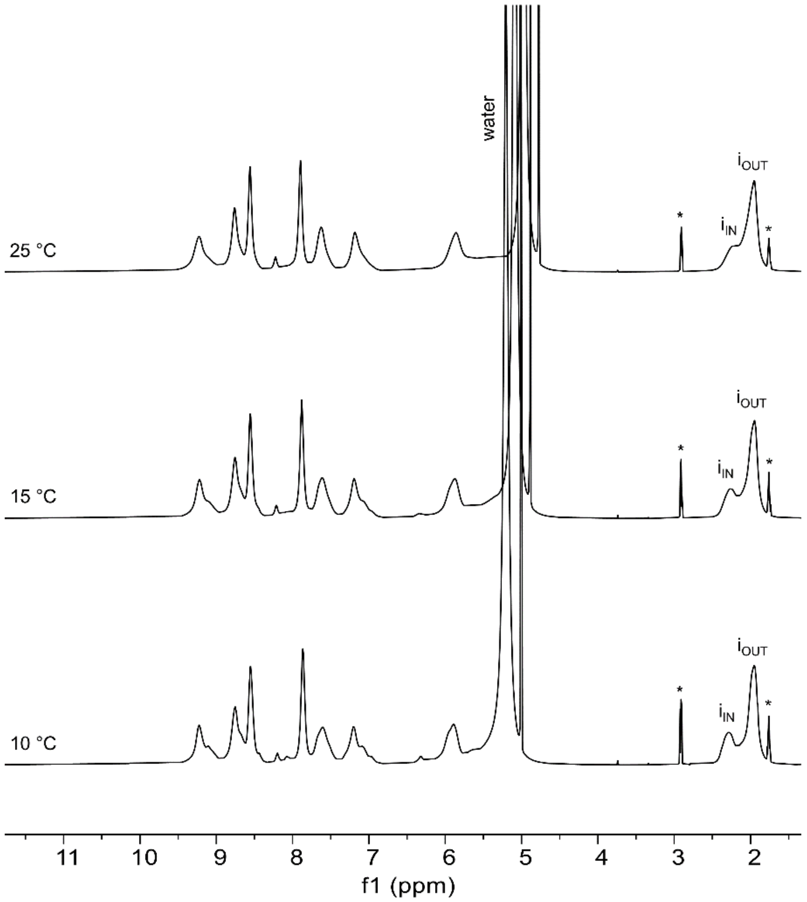

Figure S11. ^{1}H NMR (800 MHz, D_2O, referenced to DSS) of **2**[SO_4]$_4$ at 25 °C, 15 °C, and 10 °C. Signals marked with * are from DSS.

Table S2. Chemical shift of H_i *vs.* temperature.

Temperature (°C):	$H_{i\text{–}IN}$ (ppm):	$H_{i\text{–}OUT}$ (ppm):	Difference (ppm):
25	2.21	1.95	0.26
15	2.26	1.95	0.31
10	2.28	1.95	0.33

Table S3. Integrals of $H_{i\text{-}IN}$ and $H_{i\text{-}OUT}$ *vs.* temperature.

Temperature (°C):	$H_{i\text{–}IN}$ (integral):	$H_{i\text{–}OUT}$ (integral):
25	3.2	8.8
15	2.8	9.2
10	2.7	9.3

S5 UV-Vis calibration data

S5.1 Procedure

To prepare the calibration curves for cages Me-**1**[NMe_4]$_4$ and **2**[SO_4]$_4$ in water, a stock solution was prepared in a volumetric flask; the stock solution was diluted by 4/5 with water; and the UV-Vis spectrum was recorded at each concentration (S5.2 and S5.4, respectively). The calibration curves for cages Me-**1**[DDA]$_4$ in 1-butanol and **2**[$B(C_6F_5)_4$]$_8$ in ethyl acetate were also recorded. To prepare the calibration curve for cage **2**[$B(C_6F_5)_4$]$_8$ in ethyl acetate, equal volumes of cage **2**[SO_4]$_4$ in water and ethyl acetate were combined to create a biphasic system. A slight excess of $LiB(C_6F_5)_4$ (10 equiv.) was added as a concentrated stock solution in ethyl acetate, and the samples were inverted several times to promote complete phase transfer from water into the ethyl acetate layer. The ethyl acetate layer was collected, diluted by 3.5/5 with ethyl acetate, and the UV-Vis spectrum was recorded at each concentration (S5.5).

As cage Me-**1**[DDA]$_4$ begins precipitating out of 1-butanol shortly after phase transfer, the procedure for preparing the calibration curve for Me-**1**[DDA]$_4$ in 1-butanol was revised accordingly. Instead of using serial dilutions, stock solutions of Me-**1**[NMe_4]$_4$ in water were prepared at five different concentrations. Each stock solution was then used to create a biphasic system containing Me-**1**[NMe_4]$_4$ in water (5 mL) and 1-butanol (4 mL), wherein the 1-butanol contained slightly more didodecyldimethylammonium bromide (DDABr) than required for complete phase transfer (244 equiv.). An aliquot of the 1-butanol layer was diluted by 4/5 with 1-butanol, which was found to minimize scattering within the sample, and the UV-Vis spectrum was recorded (S5.3). This process was performed using one stock solution at a time, to minimize the amount of time between phase transfer and UV-Vis measurement.

S5.2 Calibration of cage Me-1[NMe_4]$_4$ in water

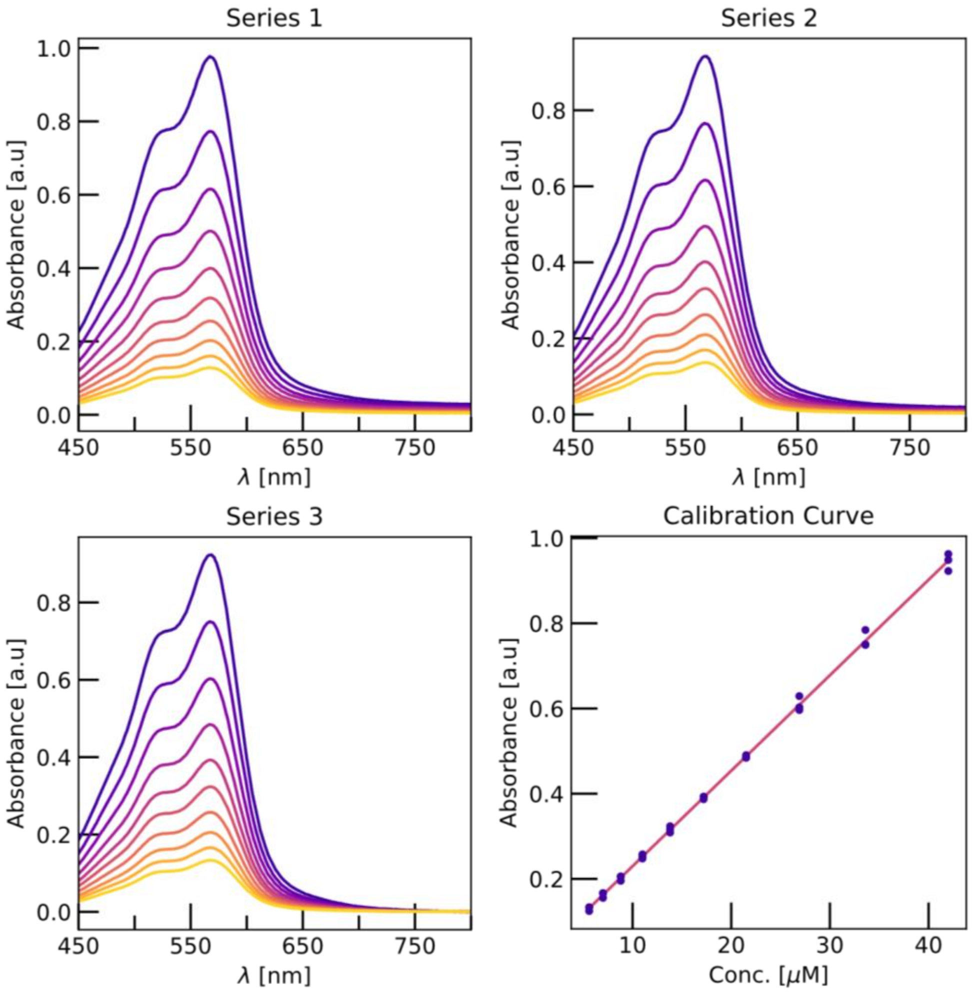

Figure S12. Raw UV-Vis data and calibration curve for cage Me-**1**[NMe_4]$_4$ in water. Abs = 0.0224C + 0.006, R^2 = 0.999.

Table S4. Summary of UV-Vis calibration data for cage Me-**1**[NMe_4]$_4$ in water.

**Concentration (μM)**	**Absorbance at λ = 567 nm**		
	Series 1	Series 2	Series 3
42.0	0.949	0.923	0.963
33.6	0.749	0.751	0.784
26.9	0.596	0.603	0.629
21.5	0.485	0.485	0.491
17.2	0.387	0.393	0.393
13.8	0.309	0.324	0.317
11.0	0.248	0.258	0.253
8.81	0.196	0.206	0.206
7.05	0.155	0.166	0.167
5.64	0.125	0.134	0.133

S5.3 Calibration of cage Me-1[DDA]$_4$ in 1-butanol

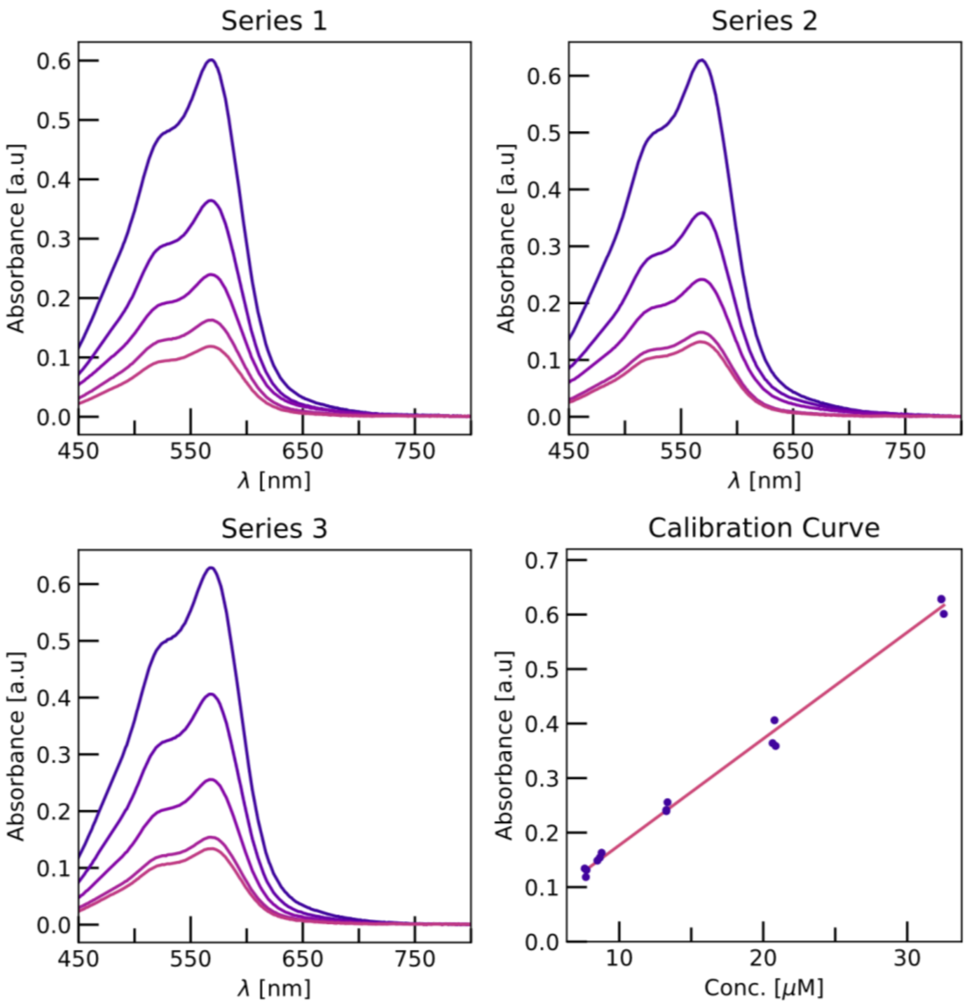

Figure S13. Raw UV-Vis data and calibration curve for cage Me-**1**[DDA]$_4$ in 1-butanol. Abs = 0.01998C – 0.0184, R^2 = 0.999.

Table S5. Summary of UV-Vis calibration data for cage Me-**1**[DDA]$_4$ in 1-butanol.

Series 1		Series 2		Series 3	
Conc. (μM)	Abs. at λ = 567 nm	Conc. (μM)	Abs. at λ = 567 nm	Conc. (μM)	Abs. at λ = 567 nm
32.5	0.601	32.4	0.628	32.4	0.629
20.7	0.364	20.9	0.359	20.8	0.406
13.3	0.240	13.3	0.242	13.4	0.255
8.77	0.163	8.48	0.149	8.65	0.154
7.68	0.119	7.72	0.131	7.60	0.134

S5.4 Calibration of cage 2[SO_4]$_4$ in water

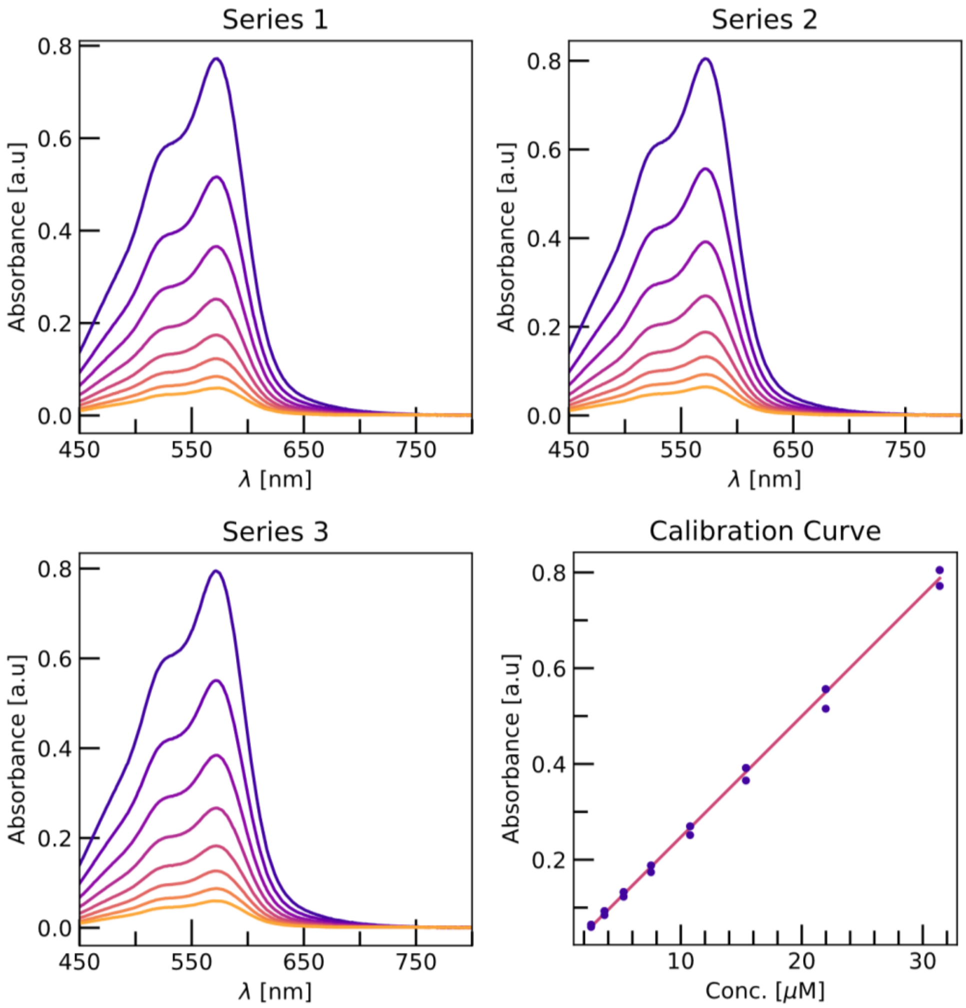

Figure S14. Raw UV-Vis data and calibration curve for cage **2**[SO_4]$_4$ in water. Abs = 0.0252C – 0.00547, R^2 = 0.998.

Table S6. Summary of UV-Vis calibration data for cage **2**[SO_4]$_4$ in water.

**Concentration (μM):**	**Absorbance at λ = 571 nm [a.u.]**		
	Series 1	Series 2	Series 3
31.4	0.772	0.805	0.805
22.0	0.515	0.556	0.556
15.4	0.365	0.392	0.392
10.8	0.251	0.270	0.270
7.54	0.174	0.188	0.188
5.28	0.123	0.132	0.132
3.69	0.084	0.093	0.093
2.59	0.060	0.064	0.064

S5.5 Calibration of cage 2[B(C_6F_5)$_4$]$_8$ in ethyl acetate

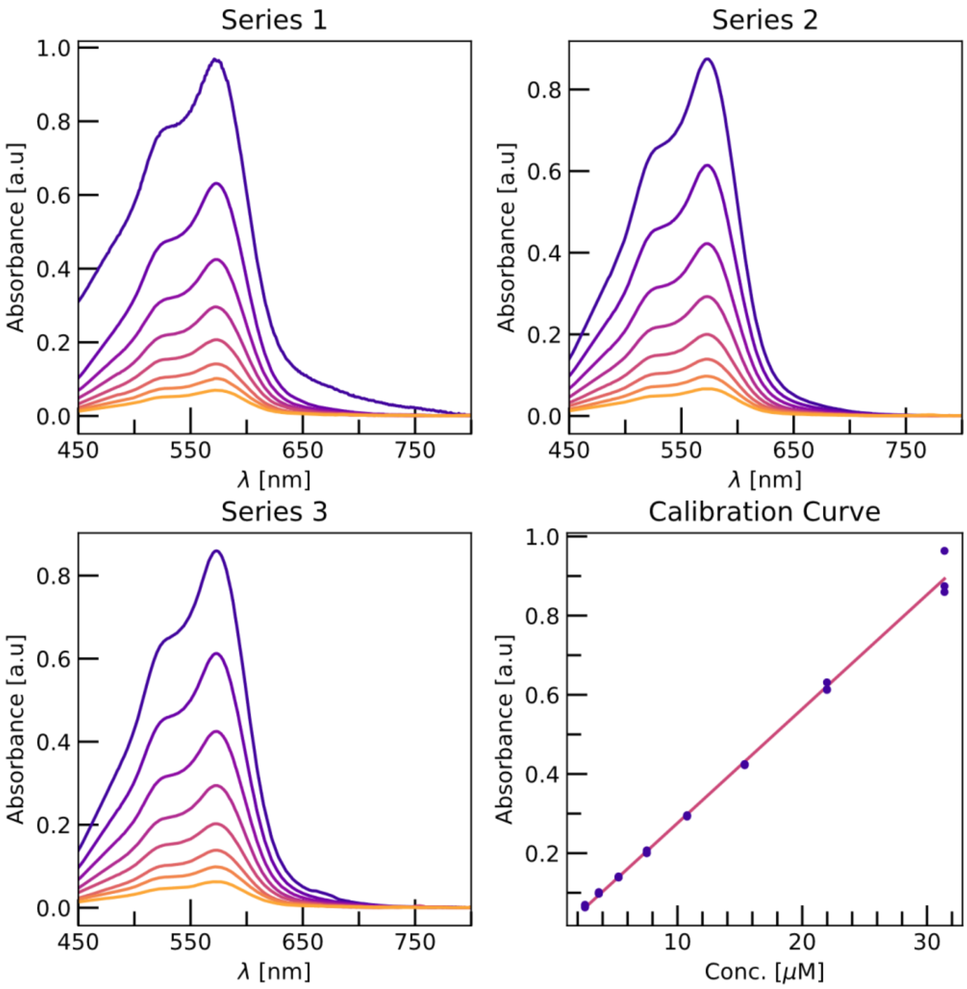

Figure S15. Raw UV-Vis data and calibration curve for cage **2**[B(C_6F_5)$_4$]$_8$ in ethyl acetate. Abs = 0.0288C – 0.0124, R^2 = 0.996.

Table S7. Summary of UV-Vis calibration data for cage **2**[B(C_6F_5)$_4$]$_8$ in ethyl acetate.

**Concentration (μM)**	**Absorbance at λ = 573 nm [a.u.]**		
	Series 1	Series 2	Series 3
31.4	0.964	0.874	0.860
22.0	0.631	0.614	0.612
15.4	0.425	0.422	0.425
10.8	0.296	0.293	0.294
7.54	0.207	0.200	0.202
5.28	0.141	0.139	0.139
3.69	0.102	0.097	0.099
2.59	0.070	0.067	0.063

S6 Stability of cages Me-1 and 2

S6.1 Procedure and summary of results

UV-Vis measurements were conducted to determine the stability of cage Me-**1**[NMe_4]$_4$ in water, cage Me-**1**[DDA]$_4$ in 1-butanol, cage **2**[SO_4]$_4$ in water, and cage **2**[$B(C_6F_5)_4$]$_8$ in ethyl acetate over 60 minutes at similar concentrations to those used for the UV-Vis measurements in sections S7, S8, and S9. Solutions in water, 1-butanol, and ethyl acetate were prepared as described in section S5. For the solutions in water and ethyl acetate, UV-Vis spectra were collected every 10 minutes over the course of 60 minutes, the maximum duration of a complete set of phase transfer experiments. For the sample containing Me-**1**[DDA]$_4$ in 1-butanol, the sample was diluted by 4/5 with 1-butanol prior to measuring the initial UV-Vis spectrum. This dilution served to reduce scattering in the sample.

The following table summarizes the proportion of cage intact after 60 minutes:

Table S8. Proportion of intact cages at UV-Vis concentrations after 60 min.

Sample	Solvent	Cage proportion (%)
Cage Me-**1**[NMe_4]$_4$	water	98.7
Cage Me-**1**[DDA]$_4$	1-butanol	94.8
Cage **2**[SO_4]$_4$	water	100
Cage **2**[$B(C_6F_5)_4$]$_8$	ethyl acetate	98.5

S6.2 Stability of cage Me-1[NMe$_4$]$_4$ in water

Table S9. Summary of UV-Vis data to measure the stability of cage Me-**1**[NMe_4]$_4$ in water.

Time (min)	Abs at λ = 567 nm	Cage Me-1[NMe_4]$_4$ (μM)	Cage proportion (%)
0	0.962	42.7	100
10	0.957	42.5	99.5
20	0.953	42.3	99.0
30	0.952	42.3	99.0
40	0.953	42.3	99.0
50	0.951	42.2	98.8
60	0.950	42.1	98.7

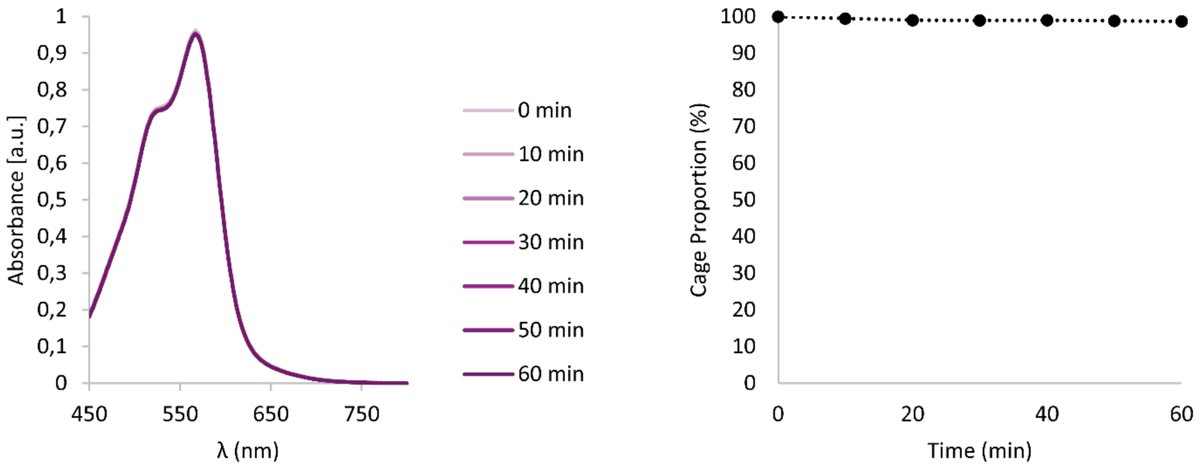

Figure S16. Stability of cage Me-**1**[NMe_4]$_4$ in water over 60 minutes.

S6.3 Stability of cage Me-1[DDA]$_4$ in 1-butanol

Table S10. Summary of UV-Vis data to measure the stability of cage Me-**1**[DDA]$_4$ in 1-butanol.

Time (min)	Abs at λ = 567 nm	Cage Me-1[DDA]$_4$ (μM)	Cage proportion (%)
0	0.807	41.1	100
10	0.801	40.8	99.3
20	0.797	40.6	98.8
30	0.787	40.1	97.6
40	0.782	39.9	97.0
50	0.771	39.3	95.7
60	0.765	39.0	94.9

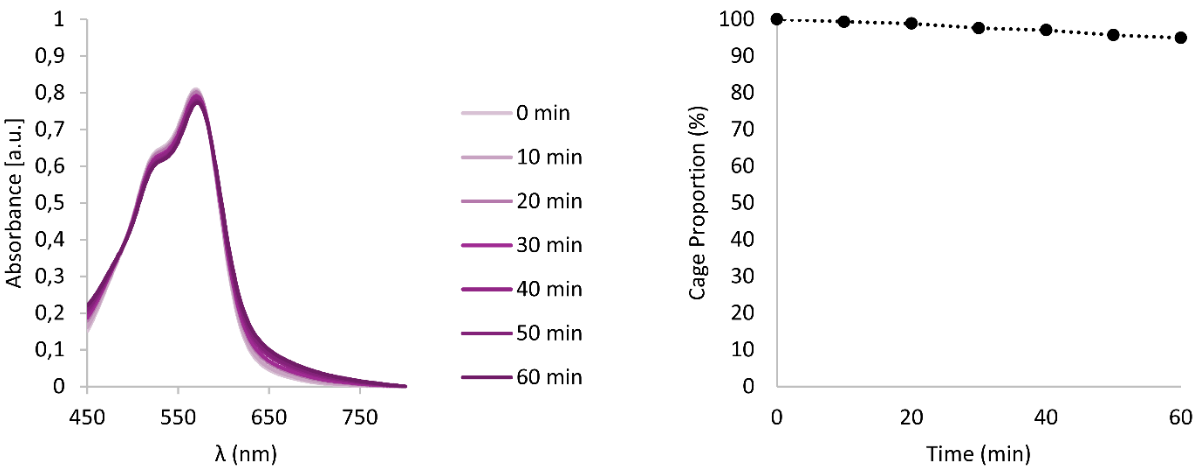

Figure S17. Stability of cage Me-**1**[DDA]$_4$ in 1-butanol over 60 minutes.

S6.4 Stability of cage 2[SO_4]$_4$ in water

Table S11. Summary of UV-Vis data to measure the stability of cage **2**[SO_4]$_4$ in water.

Time (min)	Abs at λ = 571 nm	Cage 2[SO_4]$_4$ (μM)	Cage proportion (%)
0	0.932	37.2	100
10	0.932	37.2	100
20	0.931	37.2	100
30	0.931	37.2	100
40	0.930	37.1	100
50	0.930	37.1	100
60	0.927	37.0	100

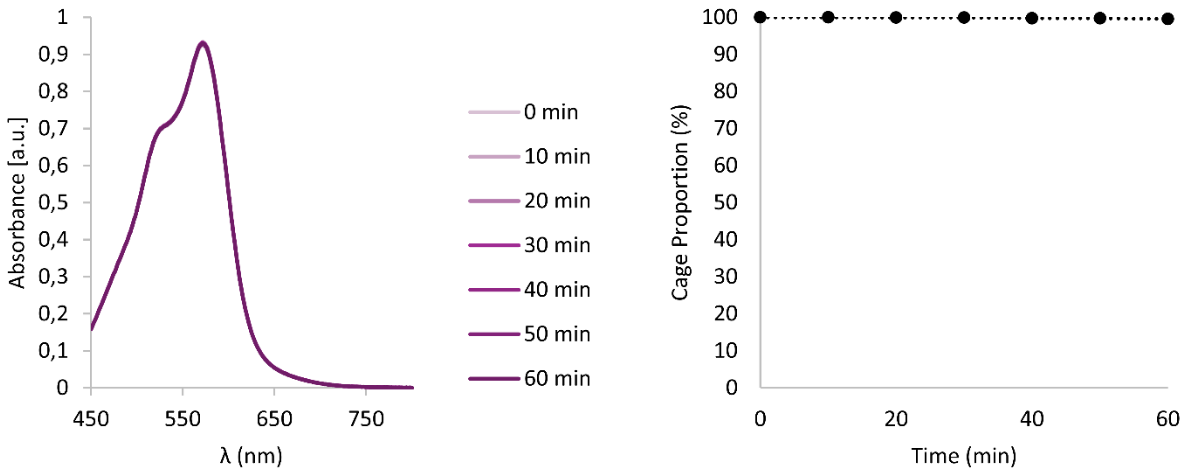

Figure S18. Stability of cage **2**[SO_4]$_4$ in water over 60 minutes.

S6.5 Stability of cage 2[B(C_6F_5)$_4$]$_8$ in ethyl acetate

Table S12. Summary of UV-Vis data to measure the stability of cage **2**[B(C_6F_5)$_4$]$_8$ in water.

Time (min)	Abs at λ = 573 nm	Cage 2[B(C_6F_5)$_4$]$_8$ (μM)	Cage proportion (%)
0	0.783	34.9	100
10	0.782	34.8	99.8
20	0.779	34.7	99.5
30	0.776	34.6	99.1
40	0.773	34.5	98.8
50	0.772	34.4	98.7
60	0.771	34.3	98.5

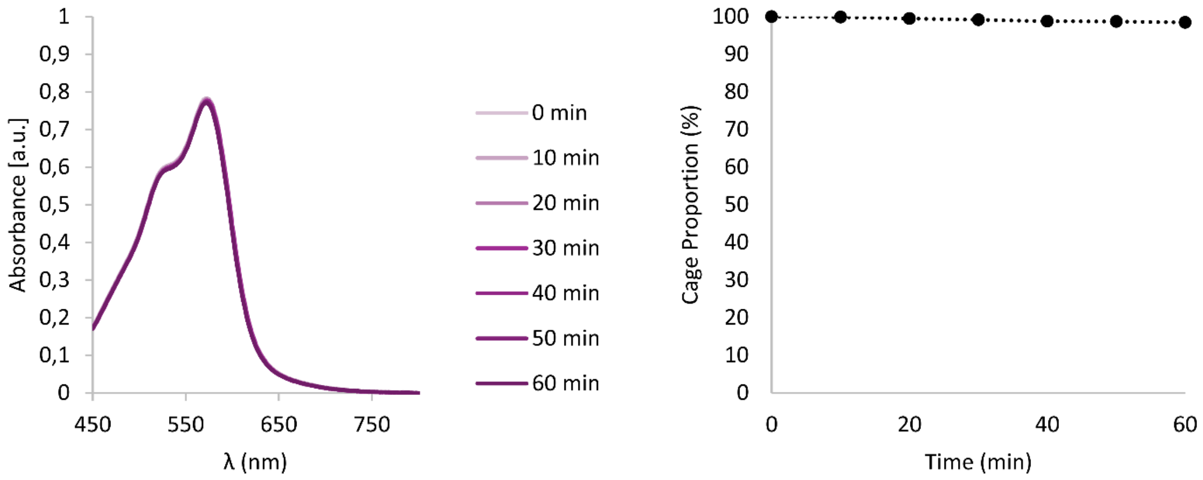

Figure S19. Stability of cage **2**[B(C_6F_5)$_4$]$_8$ in ethyl acetate over 60 minutes.

S7 Phase transfer of cage 2

S7.1 Anion titration followed by UV-Vis spectroscopy

To minimize errors due to the evaporation of ethyl acetate and/or cage decomposition, both of which accumulate over time, parallel experiments were set up such that different equivalents of salt were added to individual experiments within each set. A solution of cage **2**$[SO_4]_4$ in water (25.0 µM, 4 mL) was prepared in a 15 mL centrifuge tube. $LiB(C_6F_5)_4$ was added as a concentrated stock solution in ethyl acetate (9.38 mM), and the total volume of biphasic system was corrected to 8 mL with ethyl acetate. The tubes were inverted several times, and the layers were allowed to separate. Aliquots were taken from each layer and the absorbance was measured. Lower concentrations were measured first to minimize decomposition.

Due to the solubility of ethyl acetate in water, a dilution factor was included for the water layer. For samples with absorbance lower than 0.05, the concentration is set to 0 as these values typically represent scattering rather than absorbance.

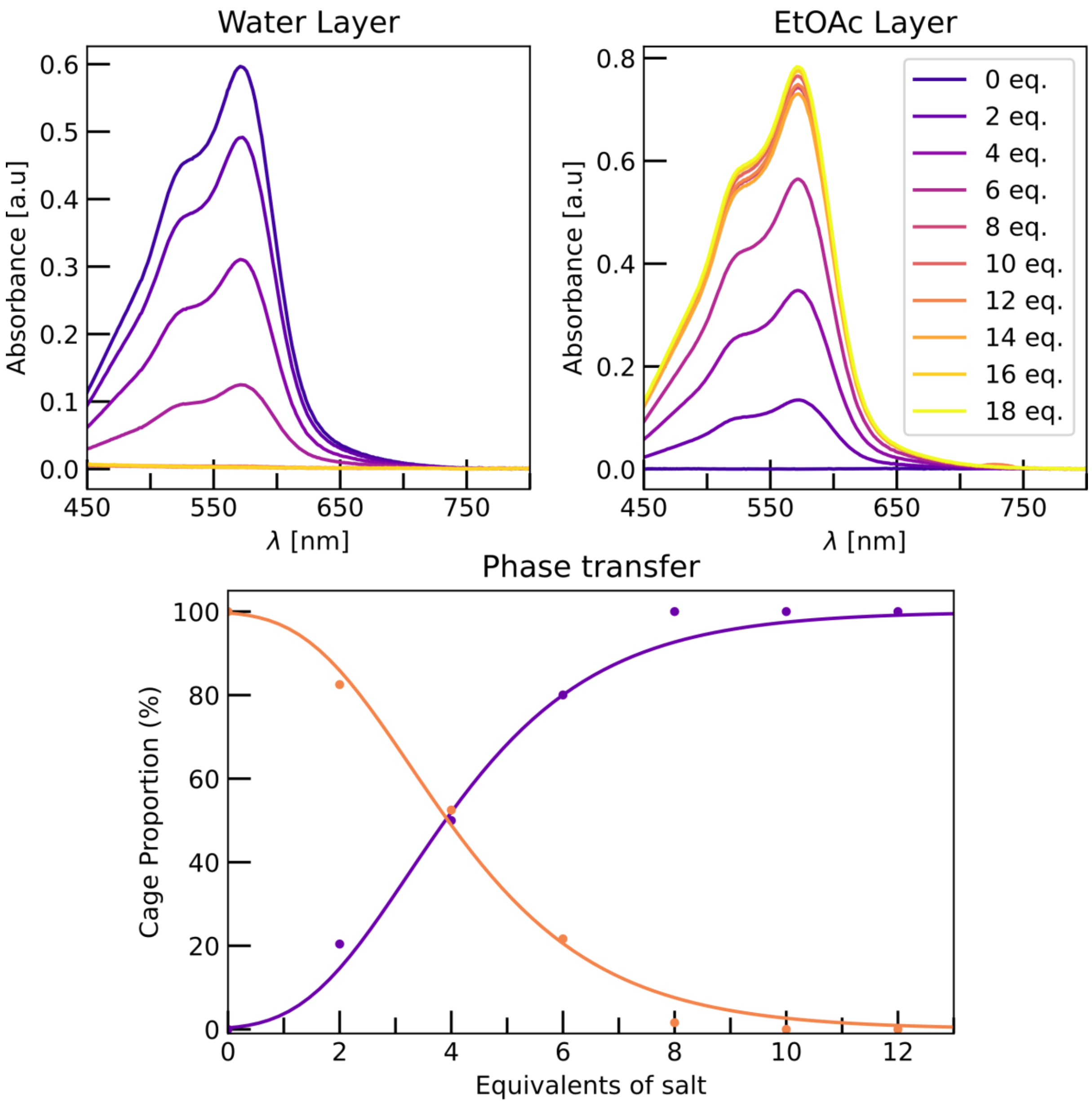

Figure S20. UV-Vis absorbance spectra and phase transfer plot for cage **2**. In the phase transfer plot, data from the water layer is depicted in orange, and data from the ethyl acetate layer is depicted in purple.

Table S13. Constants for fitted Gompertz curves in Figure S20.

**Layer**	*d*	*a*	*c*	*b*	R^2
Water	100	0	3.26	0.536	0.995
EtOAc	0	100	3.21	0.539	0.992

As $x = 3.98$ at $y = 50\%$ (S1.2), 7.95 equivalents of $LiB(C_6F_5)_4$ are needed for complete transfer.

Table S14. Summary of UV-Vis data for **2**$[SO_4]_4 \rightarrow$ **2**$[B(C_6F_5)_4]_8$.

**Layer**	**$B(C_6F_5)_4^-$ (equiv.)**	**Abs**	**Measured (μM)**	**Dilution factor**	**Corrected (μM)**	**Cage proportion (%)**
Water (λ_{max} = 572)	0	0.597	25.0	-	25.0	100
	2	0.491	23.8	1.05	20.7	82.6
	4	0.311	19.7	1.05	13.1	52.4
	6	0.125	12.5	1.05	5.4	21.7
	8	0.004	5.16	0.00	0.00	0.00
	10	0.002	0.38	0.00	0.00	0.00
	12	0.002	0.00	0.00	0.00	0.00
EtOAc (λ_{max} = 572)	0	0.0006	0.00	-	0.00	0.00
	2	0.135	5.10	-	5.10	20.4
	4	0.348	12.5	-	12.5	50.0
	6	0.565	20.0	-	20.0	80.0
	8	0.743	26.2	-	26.2	100
	10	0.765	27.0	-	27.0	100
	12	0.749	26.4	-	26.4	100

S7.2 ¹H NMR of cage 2 in ethyl acetate

To test the integrity of cage **2** following phase transfer, an aqueous solution of cage **2** in H_2O (5.0 mM, 0.75 mL) was combined with non-deuterated EtOAc (0.75 mL). A slight excess of $LiB(C_6F_5)_4$ (10 equiv.) was added as a solid to the biphasic system; the system was inverted several times; and the layers were allowed to separate. The EtOAc layer was isolated and added to an NMR tube containing a coaxial capillary with D_2O and the reference DSS. The spectrometer was locked on the deuterium signal from the capillary, and the ¹H NMR spectrum shown in Figure S21 was obtained. We only show the aromatic region of the spectrum due to the extremely large EtOAc signals in the aliphatic region.

¹H NMR (800 MHz, EtOAc, locked to D_2O in a coaxial capillary, referenced to DSS): δ_H = 9.51–9.65 (m, 12H, H_e), 9.29 (m, 12H, H_d), 9.06–9.17 (m, 12H, H_c), 8.56 (bs, 12H, H_b), 8.17–8.27 (m, 12H, H_a), 7.39–7.68 (m, 12H, H_g), 6.28–6.40 (m, 12H, H_f), 5.69–6.10 (m, 12H, H_h). $H_{i\text{-IN}}$ and $H_{i\text{-OUT}}$ were not observed due to overlap with the EtOAc signals.

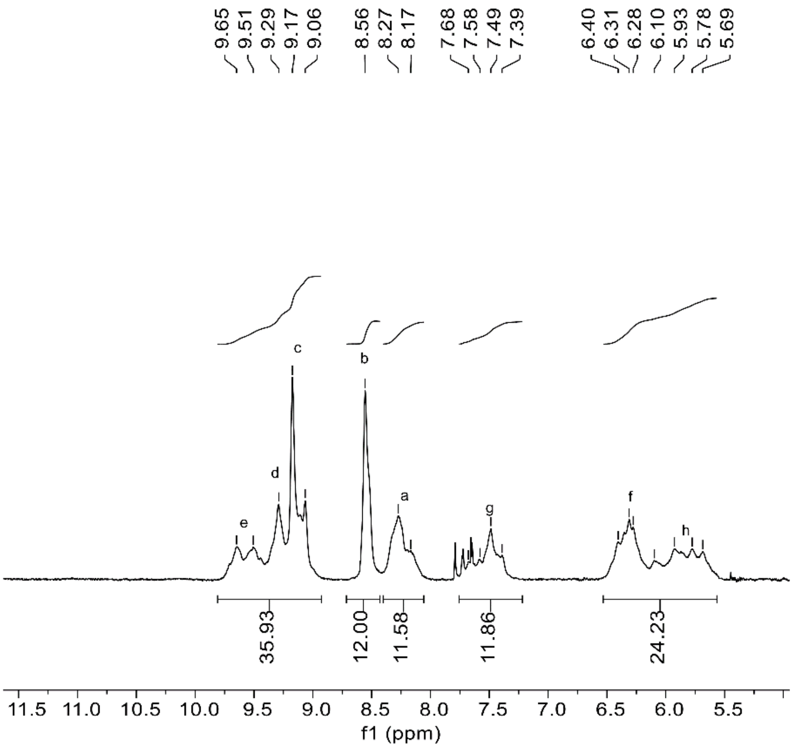

Figure S21. ¹H NMR (800 MHz, EtOAc, referenced to DSS in coaxial capillary) of cage **2**$[B(C_6F_5)_4]_8$ in non-deuterated ethyl acetate. Some signals from the unreacted amine can be observed around 7.7 ppm; these were present in the initial sample. Cage decomposition is accompanied by the emergence of an aldehyde signal, which is notably absent in this spectrum.

S7.3 ^{1}H NMR of cage 2 in water, following transfer from ethyl acetate

To 1 mL of cage **2** in D_2O (15 mM) in a 5 mL Eppendorf tube a minimum amount (10 equiv.) of a $Li(C_6F_5)_4$ solution in ethyl acetate (0.05 M) was added, then the total volume was corrected to 2 mL with ethyl acetate. The tube was inverted to promote transfer of cage **2** from water to ethyl acetate. Then the ethyl acetate layer was removed carefully, making sure to not also take some of the water layer, and added to a new tube containing a small amount of D_2O. To this tube a solution of NMe_4Br in D_2O (0.5 M) was added dropwise until no more phase transfer was observed. A sample from this water layer was collected and measured immediately by ^{1}H NMR. The resulting spectrum is presented in Figure S22. We only show the aromatic region of the spectrum due to the extremely large signals from solvent and salt in the aliphatic region.

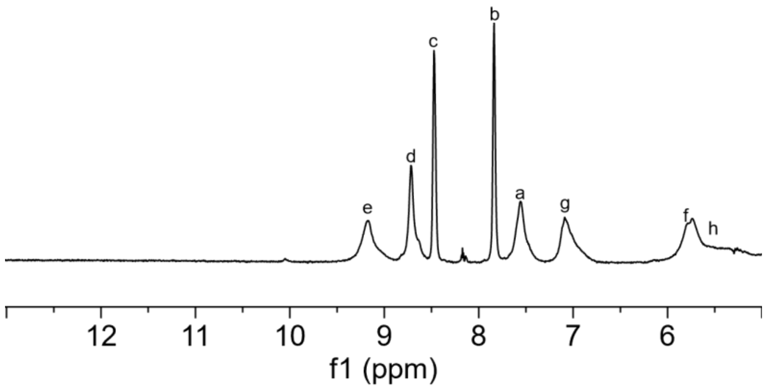

Figure S22. ^{1}H NMR (800 MHz, D_2O, referenced to DSS in coaxial capillary) of cage **2**$[Br]_8$ in D_2O. A very small aldehyde signal can be seen above 10 ppm, corresponding to breakdown of ~1% of the cage.

S8 Phase transfer of cage Me-1

S8.1 Cation titration followed by UV-Vis spectroscopy

S8.1.1 Procedure and summary of results

To minimize errors due to cage decomposition, which accumulates over time, parallel experiments were set up such that different equivalents of salt were added to individual experiments within each set. Stock solutions of cage Me-**1**[NMe_4]$_4$ were prepared at three concentrations (25, 50 and 200 µM) by dissolving solid cage in water. Aliquots (3 mL) of this stock solution were transferred to 15 mL centrifuge tubes. To these tubes, 1 mL of 1-butanol was added; DDABr was added as a stock solution in 1-butanol (500 mM); the total volume of the biphasic system was corrected to 6 mL with 1-butanol; and the tube was inverted several times. Separation of the layers was facilitated by centrifuging at 3000 RPM for 5 – 10 minutes; samples with less salt required less centrifugation. For all experiments, aliquots from the 1-butanol layer were diluted by 2/2.5 with 1-butanol. For the set of experiments corresponding to an initial concentration of 200 µM, aliquots from the water layer were diluted by 0.5/2.5 with water to reduce the maximum absorbance. To minimize decomposition, the UV-Vis spectrum from samples with lower cage concentrations were measured before samples with higher cage concentration; and samples in 1-butanol were measured before samples in water.

Table S15. Summary of concentration dependence of phase transfer of cage Me-**1**[NMe_4]$_4$→ Me-**1**[DDA]$_4$.

Concentration (µM)	Equiv.
25	123
50	107
200	42.1

*S8.1.2 Phase transfer of cage Me-**1**, 25 µM*

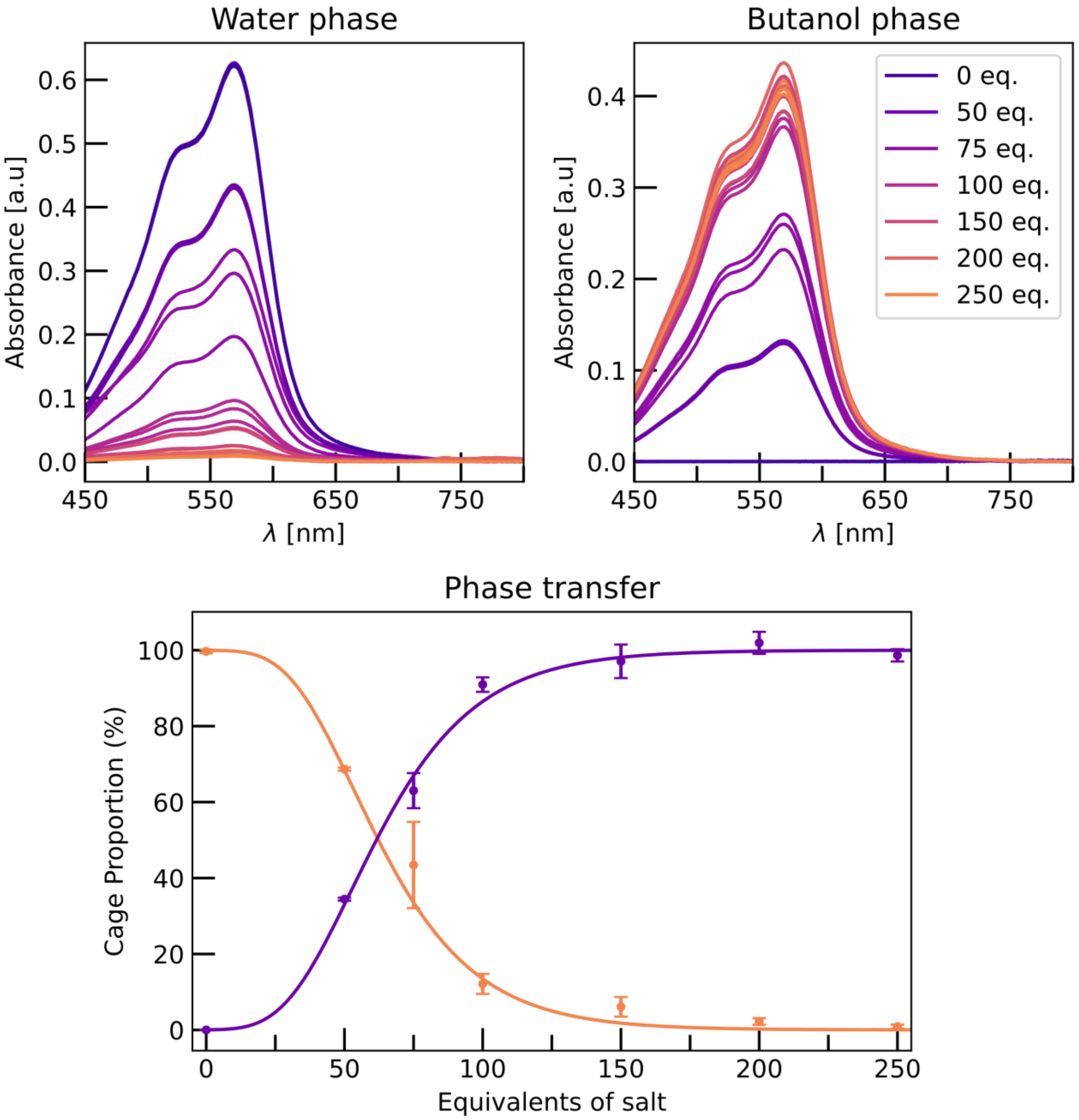

Figure S23. UV-Vis absorbance spectra and phase transfer plot for cage Me-**1** with a cage concentration of 25 µM. In the phase transfer plot, data from the water layer is depicted in yellow, and data from the 1-butanol layer is depicted in purple.

Table S16. Constants for fitted Gompertz curves in Figure S22.

**Layer**	*d*	*a*	*c*	*b*	R^2
Water	100	0	53.4	0.0415	0.996
1-Butanol	0	100	52.5	0.0407	0.991

Table S17. Summary of UV-Vis data for Me-**1**[NMe_4]$_4$ → Me-**1**[DDA]$_4$ at 25μM.

**Layer**	**DDA$^+$ (equiv.)**	**Abs**	**Measured (μM)**	**Dilution factor**	**Corrected (μM)**	**Cage proportion (%)**
Water (λ_{max} = 569)	0	0.627	27.7	-	27.7	100
	50	0.432	19.0	-	19.0	68.6
	75	0.333	14.6	-	14.6	52.7
	100	0.064	2.59	-	2.59	9.34
	150	0.054	2.14	-	2.14	7.75
	200	0.026	0.890	-	0.890	3.22
	250	0.015	0.413	-	0.413	1.45
	0	0.621	27.5	-	27.5	99.1
	50	0.430	18.9	-	18.9	68.3
	75	0.296	13.0	-	13.0	46.8
	100	0.096	4.04	-	4.04	14.6
	150	0.025	0.867	-	0.867	3.13
	200	0.153	0.423	-	0.423	1.53
	250	0.005	0.140	-	0.140	0.505
	0	0.626	27.7	-	27.7	99.9
	50	0.435	19.2	-	19.2	69.1
	75	0.197	8.53	-	8.53	30.8
	100	0.083	3.46	-	3.46	12.5
	150	0.052	2.06	-	2.06	7.42
	200	0.018	0.537	-	0.537	1.94
	250	0.009	0.173	-	0.173	0.626
1-Butanol (λ_{max} = 569)	0	0.0004	0	1.25	0	0
	50	0.130	7.58	1.25	9.47	34.2
	75	0.260	14.3	1.25	17.8	64.2
	100	0.383	20.6	1.25	25.7	92.8
	150	0.400	21.4	1.25	26.8	96.6
	200	0.420	22.5	1.25	28.0	101
	250	0.416	22.2	1.25	27.8	100
	0	0.0003	0	1.25	0	0
	50	0.133	7.73	1.25	9.67	34.9
	75	0.232	12.8	1.25	16.0	57.9
	100	0.367	19.7	1.25	24.6	89.0
	150	0.422	22.5	1.25	28.2	101
	200	0.436	23.3	1.25	29.1	105
	250	0.402	21.5	1.25	26.9	97.2
	0	0.0004	0	1.25	0	0
	50	0.130	7.58	1.25	9.48	34.2
	75	0.271	14.8	1.25	18.5	66.9
	100	0.376	20.2	1.25	25.2	91.1
	150	0.384	20.6	1.25	25.7	92.9
	200	0.412	22.0	1.25	27.5	99.4
	250	0.407	21.8	1.25	27.2	98.4

*S8.1.3 Phase transfer of cage Me-**1**, 50 µM*

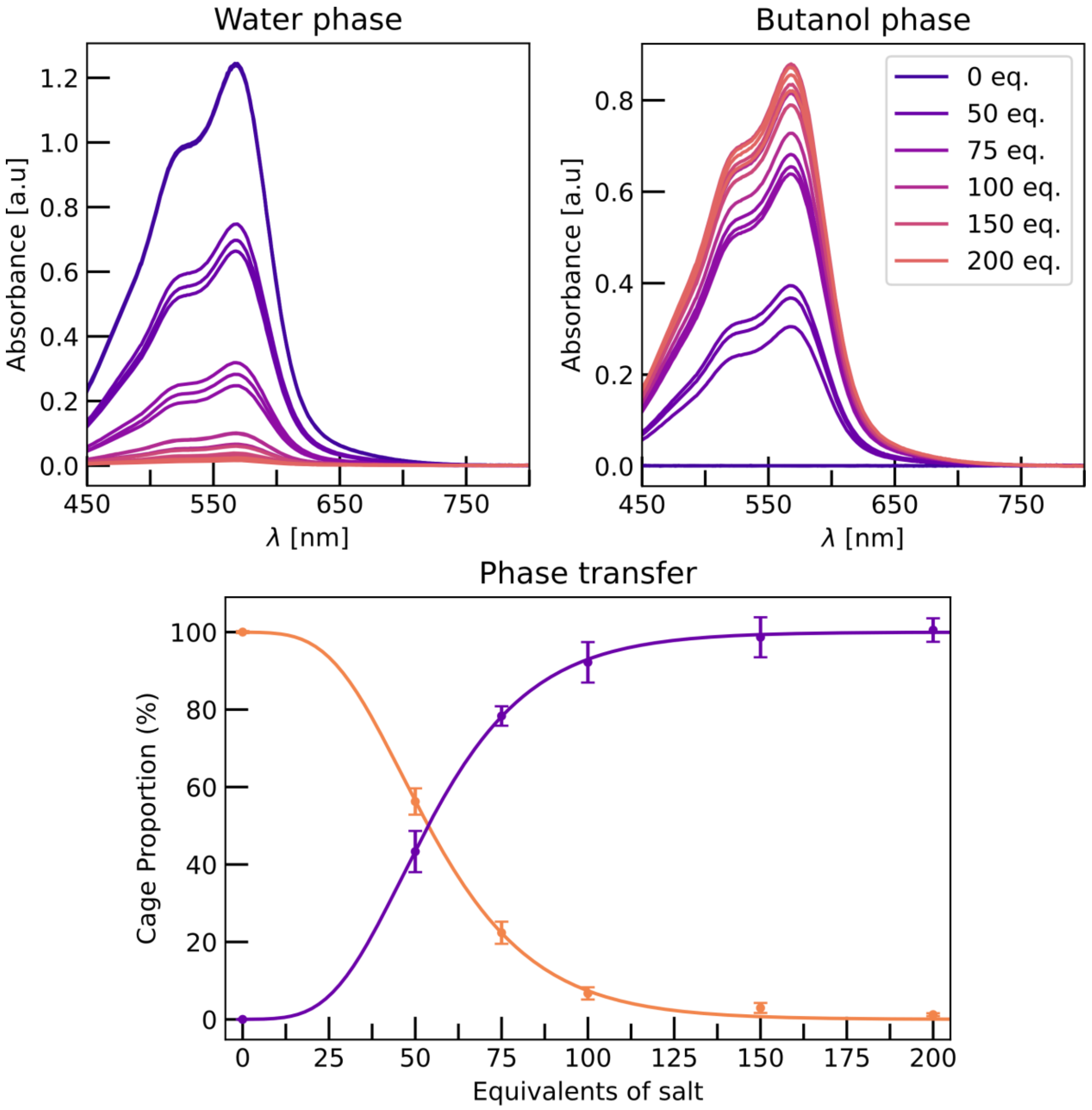

Figure S24. UV-Vis absorbance spectra and phase transfer plot for cage Me-**1** with a cage concentration of 50 µM. In the phase transfer plot, data from the water layer is depicted in yellow, and data from the 1-butanol layer is depicted in purple.

Table S18. Constants for fitted Gompertz curves in Figure S23.

**Layer**	*d*	*a*	*c*	*b*	R^2
Water	100	0.00	46.1	0.0476	0.997
1-Butanol	0.00	100	46.2	0.0487	0.992

Table S19. Summary of UV-Vis data for Me-**1**$[NMe_4]_4$ → Me-**1**$[DDA]_4$ at 50μM.

Layer	DDA^+ (equiv.)	Abs	Measured (μM)	Dilution factor	Corrected (μM)	Cage proportion (%)
Water (λ_{max} = 568)	0	1.24	55.3	-	55.3	100
	50	0.747	33.1	-	33.1	60.0
	75	0.318	14.0	-	14.0	25.3
	100	0.0663	2.70	-	2.70	4.90
	150	0.0380	1.44	-	1.44	2.61
	200	0.0158	0.447	-	0.447	0.811
	0	1.24	55.1	-	55.1	100
	50	0.664	29.4	-	29.4	53.3
	75	0.282	12.3	-	12.3	22.4
	100	0.0993	4.18	-	4.18	7.58
	150	0.0291	1.04	-	1.04	1.89
	200	0.239	0.810	-	0.810	1.47
	0	1.24	55.0	-	55.0	99.8
	50	0.697	30.8	-	30.8	56.0
	75	0.248	10.8	-	10.8	19.6
	100	0.101	4.25	-	4.25	7.72
	150	0.0601	2.42	-	2.42	4.40
	200	0.0233	0.784	-	0.784	1.42
1-Butanol (λ_{max} = 567)	0	0.0008	0	1.25	0	0
	50	0.305	16.6	1.25	20.7	27.6
	75	0.638	33.6	1.25	42.0	76.3
	100	0.815	42.7	1.25	53.4	96.9
	150	0.833	43.6	1.25	54.1	98.9
	200	0.856	22.8	1.25	55.9	101
	0	0.0007	0	1.25	0	0
	50	0.395	21.2	1.25	26.4	48.0
	75	0.654	33.4	1.25	43.1	78.1
	100	0.790	41.4	1.25	51.7	93.9
	150	0.878	45.9	1.25	57.4	104
	200	0.820	43.0	1.25	53.7	97.5
	0	0.0006	0	1.25	0	0
	50	0.368	19.8	1.25	24.7	44.8
	75	0.681	35.8	1.25	44.8	81.3
	100	0.728	38.2	1.25	47.8	86.7
	150	0.789	41.3	1.25	51.7	93.8
	200	0.872	45.6	1.25	57.0	103

*S8.1.4 Phase transfer of cage Me-**1**, 200 µM*

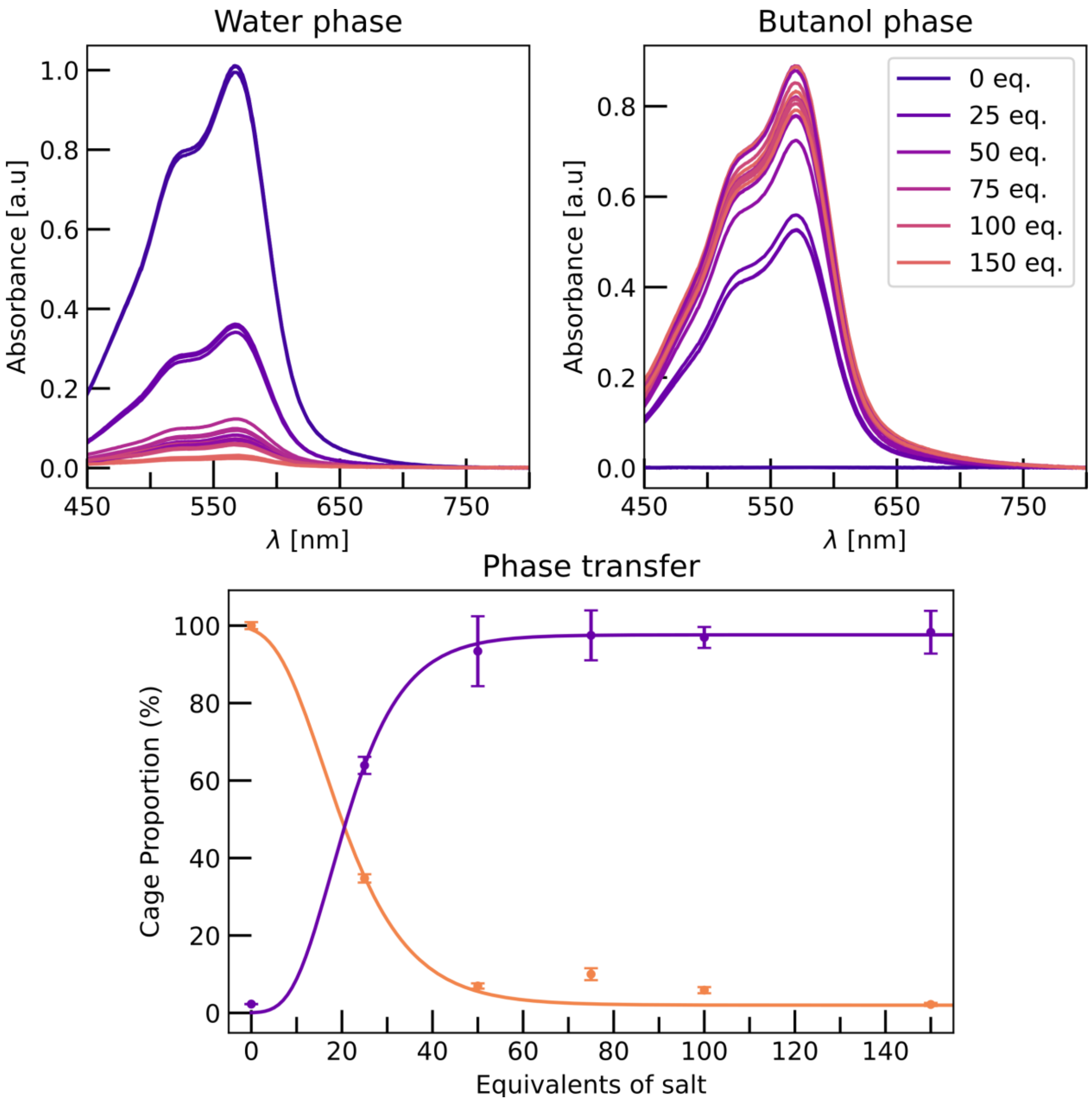

Figure S25. UV-Vis absorbance spectra and phase transfer plot for cage Me-**1** with a cage concentration of 200 µM. In the phase transfer plot, data from the water layer is depicted in yellow, and data from the 1-butanol layer is depicted in purple.

Table S20. Constants for fitted Gompertz curves in Figure S24.

**Layer**	*d*	*a*	*c*	*b*	R^2
Water	100	2	15.8	0.0965	0.997
1-Butanol	0	97.6	17.6	0.116	0.992

Table S21. Summary of UV-Vis data for Me-**1**[NMe_4]$_4$ → Me-**1**[DDA]$_4$ at 200 µM.

Layer	DDA^+ (equiv.)	Abs	Measured (µM)	Dilution factor	Corrected (µM)	Cage proportion (%)
Water (λ_{max} = 568)	0	1.24	55.3	-	55.3	100
	50	0.747	33.1	-	33.1	60.0
	75	0.318	14.0	-	14.0	25.3
	100	0.0663	2.70	-	2.70	4.90
	150	0.0380	1.44	-	1.44	2.61
	0	1.24	55.1	-	55.1	100
	50	0.664	29.4	-	29.4	53.3
	75	0.282	12.3	-	12.3	22.4
	100	0.0993	4.18	-	4.18	7.58
	150	0.0291	1.04	-	1.04	1.89
	0	1.24	55.0	-	55.0	99.8
	50	0.697	30.8	-	30.8	56.0
	75	0.248	10.8	-	10.8	19.6
	100	0.101	4.25	-	4.25	7.72
	150	0.0601	2.42	-	2.42	4.40
1-Butanol (λ_{max} = 570)	0	0.0012	0	5	0	0
	50	0.527	27.9	5	139	62.7
	75	0.724	38.0	5	190	85.4
	100	0.778	40.8	5	203	91.6
	150	0.851	44.5	5	223	100
	0	0.0013	0	5	0	0
	50	0.525	27.9	5	139	62.6
	75	0.778	40.8	5	204	91.6
	100	0.889	46.5	5	232	104
	150	0.806	442.2	5	211	95
	0	0.0014	0	5	0	0
	50	0.559	29.6	5	148	66.5
	75	0.879	45.9	5	230	103
	100	0.805	43.0	5	215	96.6
	150	0.817	42.8	5	214	96.0

S8.2 Cation titration followed by DLS

S8.2.1 Procedure and summary of results

The mechanism by which cage Me-**1** transfers from water into 1-butanol was probed using DLS. Two parallel experiments were conducted to monitor aggregation in water and 1-butanol, in the presence and absence of the cage. Samples were prepared in 5 mL plastic Eppendorf tubes, then transferred to a quartz DLS cuvette for analysis. A solution of cage Me-**1** in water (2 mL, 75 µM) was combined with 1-butanol (2 mL), and DDABr added as a stock solution in 1-butanol (0.343 M). The tubes were inverted several times, and the layers were allowed to separate. Controls were prepared in the same way, but without cage Me-**1**. Samples were measured in order of least to most salt, alternating between water and 1-butanol layers.

Parameters for the DLS measurements are summarized below:

Material: liposomes
Dispersant: water
Method builder: size
Temperature: 298 K
Equilibration time: 0 min
Analysis model: general purpose
Number of runs: automatic
Run duration: 1.68 s
Optical settings: fluorescence filter

Samples containing cage Me-**1** were measured 3 times; controls were measured 5 times. Measurements that failed to pass the instruments quality control check, or that had number fluctuations, were discarded. As no significant trends were observed in the water layer, this data is not included.

*S8.2.2 Size of peaks 1 and 2 in the presence and absence of cage Me-**1** in 1-butanol*

Table S22. Size (nm) of particles in the absence of cage Me-**1**.

**DDA⁺ (equiv.)**	**Peak 1 (nm)**	**Peak 2 (nm)**
0.00	2.7 ± 0.0	895 ± 89
11.4	2.9 ± 0.1	1397 ± 47
22.9	3.0 ± 0.2	1925 ± 404
34.3	3.0 ± 0.1	1549 ± 74
45.7	3.1 ± 0.0	1817 ± 273
57.2	3.1 ± 0.2	2129 ± 334
68.6	3.1 ± 0.0	2183 ± 19
80.0	3.1 ± 0.5	3186 ± 817
91.5	3.0 ± 0.1	3038 ± 137
103	3.0 ± 0.3	3181 ± 859
114	3.1 ± 0.0	3210 ± 410
126	3.2 ± 0.2	2457 ± 146

Table S23. Size (nm) of particles in the presence of cage Me-**1**.

**DDA⁺ (equiv.)**	**Peak 1 (nm)**	**Peak 2 (nm)**
0.00	2.6 ± 0.1	709 ± 308
11.4	2.5 ± 0.2	1009 ± 247
22.9	2.6 ± 0.2	1717 ± 88
34.3	2.7 ± 0.2	1694 ± 271
57.2	2.6 ± 0.4	1487 ± 1270
68.6	0.1 ± 0.3	2261 ± 1360
91.5	0.0 ± 0.0	2660 ± 3076
103	0.7 ± 0.7	1794 ± 1666
114	0.8 ± 0.8	1732 ± 1448
126	0.0 ± 0.0	4698 ± 925

*S8.2.3 Integrals of peaks 1 and 2 in the presence and absence of cage Me-**1** in 1-butanol*

Table S24. Integrals of DLS peaks in the absence of cage Me-**1**.

DDA$^+$ (equiv.)	Peak 1 (integral)	Peak 2 (integral)
0.00	48.7 ± 1.6	51.3 ± 1.6
11.4	76.5 ± 1.9	23.5 ± 1.9
22.9	84.4 ± 4.9	15.6 ± 4.9
34.3	73.5 ± 1.3	26.5 ± 1.3
45.7	76.9 ± 2.5	22.9 ± 2.2
57.2	81.6 ± 3.9	18.4 ± 3.9
68.6	77.1 ± 2.1	22.9 ± 2.1
80.0	84.7 ± 3.5	10.7 ± 2.6
91.5	91.0 ± 2.1	8.48 ± 1.88
103	85.9 ± 4.6	12.3 ± 2.7
114	87.8 ± 1.1	12.2 ± 1.1
126	82.2 ± 2.8	17.7 ± 2.8

Table S25. Integrals of DLS peaks in the presence of cage Me-**1**.

DDA$^+$ (equiv.)	Peak 1 (integral)	Peak 2 (integral)
0.00	72.5 ± 4.0	18.8 ± 5.1
11.4	41.0 ± 6.3	58.6 ± 5.8
22.9	8.7 ± 6.4	83.4 ± 8.4
34.3	14.2 ± 3.9	78.1 ± 12.6
57.2	10.7 ± 4.0	69.3 ± 23.5
68.6	1.2 ± 2.7	98.8 ± 2.7
91.5	0.0 ± 0.0	100.0 ± 0.0
103	5.6 ± 5.5	94.3 ± 5.3
114	6.9 ± 7.2	93.1 ± 7.2
126	0.0 ± 0.0	100.0 ± 0.0

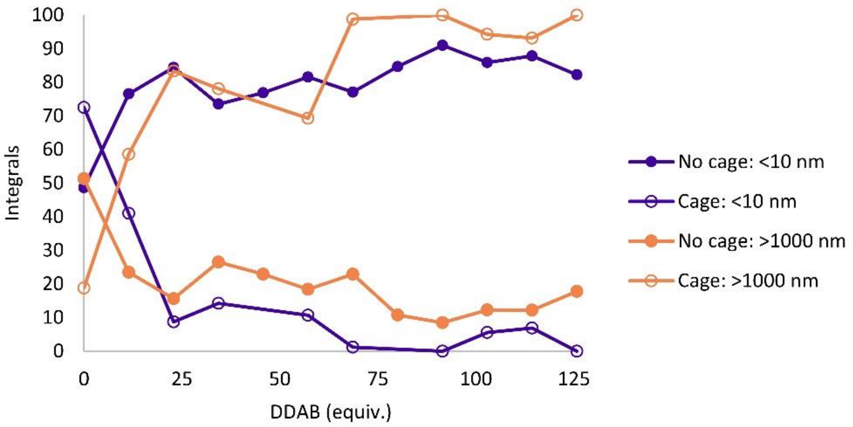

Figure S26. Plot of integrals for the DLS peaks 1 (<10 nm, purple) and 2 (>1000 nm, orange) in the presence (open circles) and absence (closed circles) of cage Me-**1**, in 1-butanol upon the addition of DDABr.

S8.2.4 Representative DLS spectra

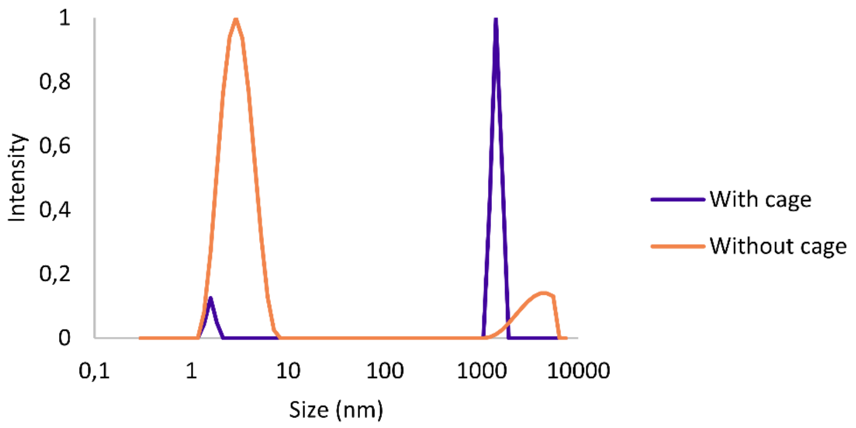

Figure S27. Representative DLS spectra of the 1-butanol layer after the addition of 120 equivalents DDABr, in the presence (purple) and absence (orange) of cage Me-**1**.

S8.3 ¹H NMR of cage Me-1 in 1-butanol

To test the integrity of cage Me-**1** following phase transfer, an aqueous solution of cage Me-**1** in H_2O (5.0 mM, 0.75 mL) was combined with a 500 mM solution of DDABr in deuterated 1-butanol (0.75 mL). The system was inverted several times; and the layers were allowed to separate. The 1-butanol layer was isolated and added to an NMR tube containing a coaxial capillary with D_2O and the reference DSS. The spectrometer was locked on the deuterium signal from the capillary, and the ^1H NMR spectrum shown in Figure S28 was obtained. We only show the aromatic region of the spectrum due to the extremely large signals from solvent and salt in the aliphatic region.

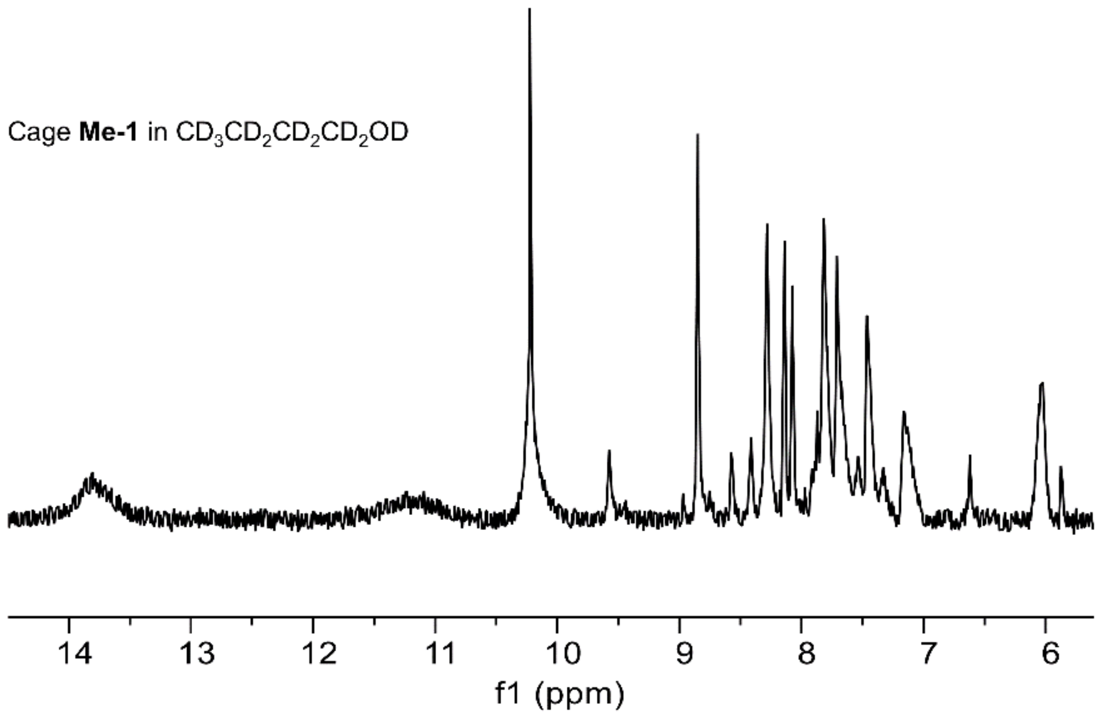

Figure S28. ^1H NMR (800 MHz, $CD_3CD_2CD_2CD_2OD$, locked to D_2O in a coaxial capillary, referenced to DSS) of cage Me-**1**[DDA]$_4$ in 1-butanol.

This spectrum contains twice the number of signals as expected for cage Me-**1**. In addition to the expected signals, a set of larger, broader signals were observed, including a signal near 14 ppm. Notably, signals characteristic of decomposition (signals from the amine or aldehyde subcomponents) were not observed; precipitation was not observed; and the solution remained the dark purple colour expected from the cage. Due to the complexity of this system, we hypothesized the spectrum represents two distinct populations of cage Me-**1**, perhaps corresponding to one population residing within salt aggregates, and one interacting strongly with 1-butanol. Exchange between these two populations would be slow on the NMR timescale, leading to two sets of signals.

To test this hypothesis, cage Me-**1** was transferred from 1-butanol back to water following the addition of tetramethylammonium bromide, which triggered cation exchange from cage Me-**1**[DDA]$_4$ back to Me-**1**[NMe$_4$]$_4$. As seen in Figure S29 on the following page, all species initially resident in the 1-butanol layer underwent phase transfer to the water layer. The ^1H NMR spectrum of the 1-butanol layer contains no signals in the aromatic region, whereas both species initially resident in the 1-butanol layer are observed in the water layer. In addition to the seven signals readily assignable to cage Me-**1**[NMe$_4$]$_4$ in water, seven additional signals (H_1 – H_7) are attributed to a second population of cage Me-**1**, still strongly interacting with 1-butanol despite having been transferred back to water. Small amounts of amine subcomponent (*) could also be observed, corresponding to a very small amount of cage degradation.

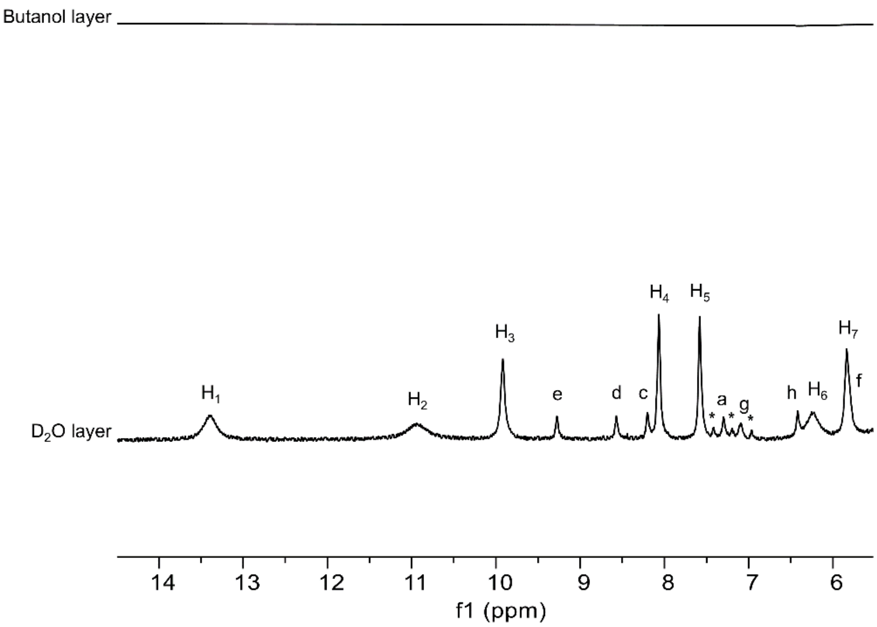

Figure S29. ^{1}H NMR (800 MHz, 298 K, referenced to DSS) spectra from the 1-butanol (top) and D_2O (bottom) layers following phase transfer of cage Me-**1** from 1-butanol to water. Spectra were collected within 15 minutes of phase transfer.

A second ^{1}H NMR spectrum of the water layer was collected the next day (Figure S30, following page), to investigate whether the relative populations of the two cage species change over time – they do not. As we hypothesized that the larger, broader set of signals arise due to interactions with 1-butanol, we also combined a fresh solution of cage Me-**1** in water with a layer of 1-butanol (with no additional salt), to test if introduction of 1-butanol triggers the formation of these signals – it does. Finally, we removed all solvent from the sample containing cage Me-**1**$[NMe_4]_4$ in water following transfer from 1-butanol back to water, redissolved the sample in D_2O, and immediately collected the ^{1}H NMR spectrum. Only one set of signals, corresponding to the expected set of ^{1}H signals from cage Me-**1**$[NMe_4]_4$, were observed. This result confirms our hypothesis that the two sets of signals observed in the 1-butanol layer following phase transfer arise from two populations of intact cage Me-**1**.

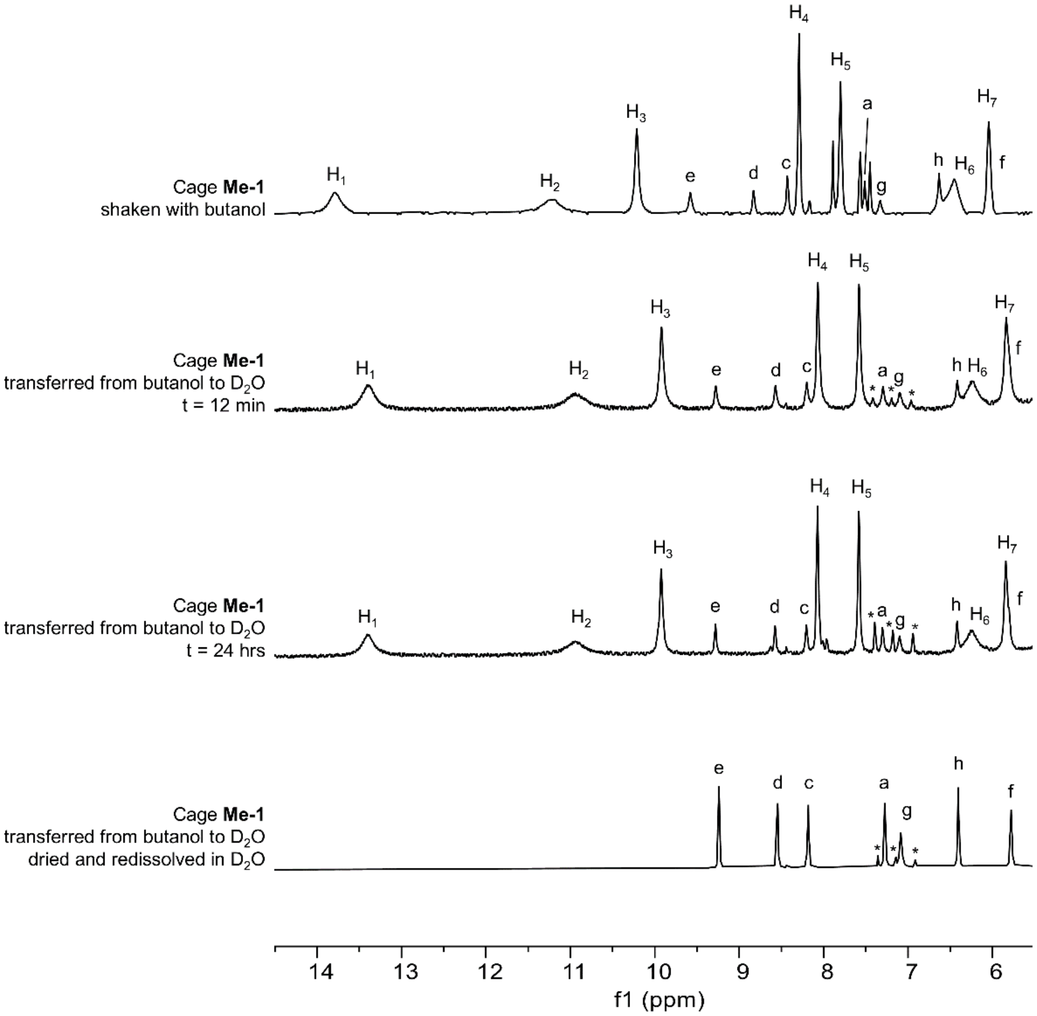

Figure S30. Stacked ^{1}H NMR (800 MHz, D_2O, 298 K, referred to DSS) spectra of cage Me-**1**$[NMe_4]_4$ in D_2O under various conditions.

S9 Orthogonal phase transfer

S9.1 ^{1}H NMR spectra of cages Me-1 and 2 over time

Solutions of cages Me-**1** and **2** in D_2O were combined to create a mixture wherein the two cages were present in equal concentrations (5.9 mM each). ^{1}H NMR spectra of this mixture were collected after 1 hr, and after 5 hrs 30 min. This timeframe is longer than the time required to complete each UV-Vis measurement described in the following section. When cages Me-**1** and **2** are combined, their ^{1}H NMR signals shift with respect to the corresponding spectra of cages Me-**1** and **2** in isolation, likely due to difference in salt composition. To facilitate comparison, the spectra below have therefore been aligned. At both time points, the ^{1}H NMR spectrum of the mixture matches the expected overlap of the spectra from cages Me-**1** and **2** individually. No ligand exchange between these two geometrically similar cages is observed.

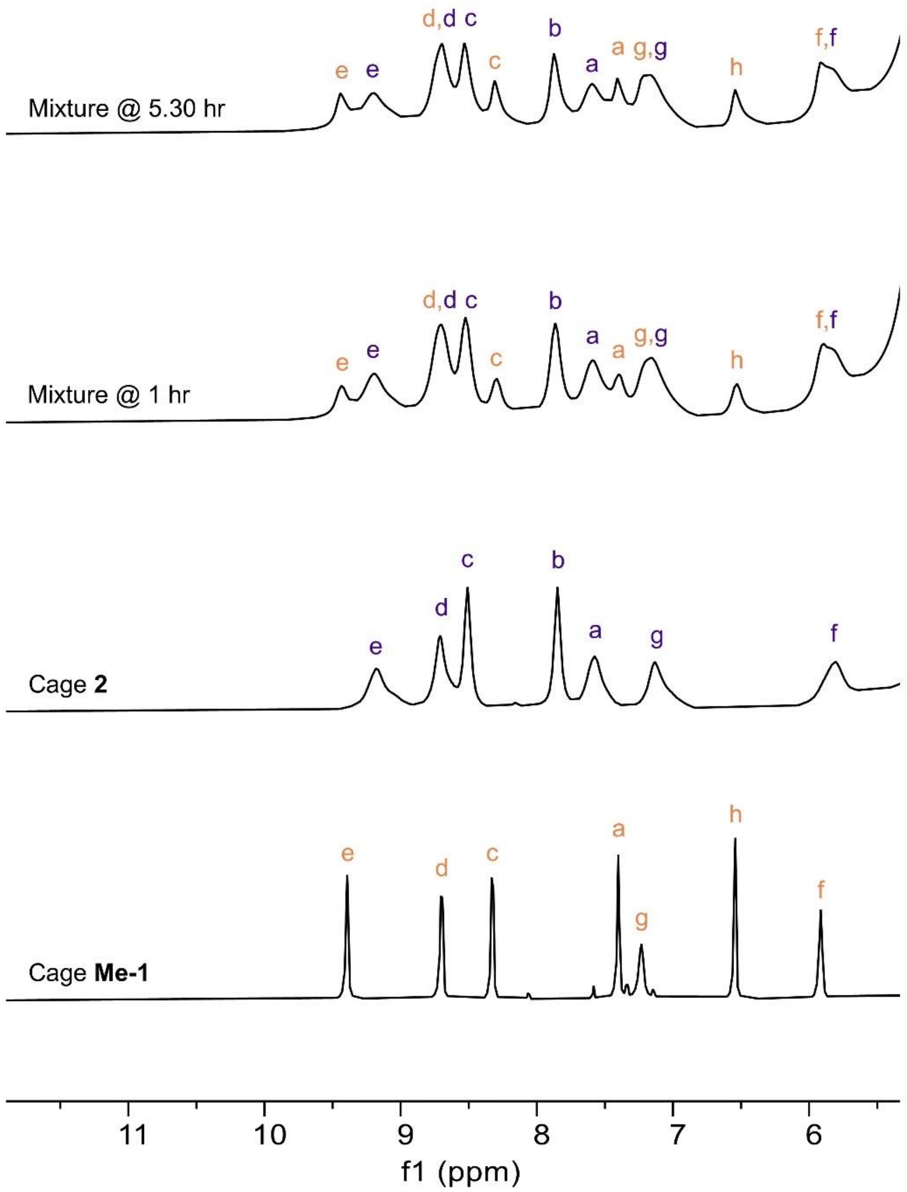

Figure S31. Stacked ^{1}H NMR (800 MHz, D_2O, 298 K, aligned) spectra of cage Me-**1**[NMe_4]$_4$, cage **2**[SO_4]$_4$, and a 50:50 mixture of cages Me-**1**[NMe_4]$_4$ and **2**[SO_4]$_4$ 1 hr and 5.30 hrs after combining two stock solutions.

S9.2 Orthogonal phase transfer followed by UV-Vis spectrometry

S9.2.1 Procedure and summary of the data

To minimize errors due to the evaporation of ethyl acetate and/or cage decomposition, both of which accumulate over time, parallel experiments were set up such that different equivalents of salt were added to individual experiments within each set. For both experiments (S9.2.2 and S9.2.3) an initial mixture containing both cage Me-**1** (25 μM) and cage **2** (25 μM) in water was prepared. Aliquots of this mixture (3 – 6 mL) were added to 15 mL centrifuge tubes. To these tubes, 1 mL of 1-butanol was added, followed by addition of the salt. DDABr was added as a stock solution in 1-butanol (500 mM), and $LiB(C_6F_5)_4$ was added as a stock solution in EtOAc (9.38 mM). The total volume of the biphasic system was then corrected such that the volume of the organic layer was equal to that of the water layer. The tubes were then inverted several times, and separation of the layers was facilitated by centrifuging at 3000 RPM for 10 minutes (S9.2.2) or 3 minutes (S9.2.3). For both sets of experiments, aliquots from the water layer were used without further dilution, while aliquots from the organic layer were diluted by 4/5 to reduce scattering. For the set of experiments involving selective transfer of cage Me-**1** (S9.2.2), the 1-butanol layer was measured first. For the set of experiments involving selective transfer of cage **2**, the water layer was measured first, as decomposition of cage Me-**1** was observed over time in the presence of $LiB(C_6F_5)_4$.

Gompertz functions were fitted to the UV-Vis data in the organic layers as these layers only contain one cage. The expected absorbance from the transferring cage in water was then calculated by subtracting its concentration in the organic layer from its initial concentration. This value was then subtracted from the total absorbance in water to determine the absorbance, and subsequently the concentration, of the non-transferring cage. For the transfer of cage **2** to ethyl acetate, x = 2.75 at y = 50%, and 5.51 equivalents of $LiB(C_6F_5)_4$ are thus required for complete transfer. For transfer of cage Me-**1** to 1-butanol, x = 235.5 at y = 50%, and thus 471 equivalents of DDABr are required for complete transfer.

*S9.2.2 Selective transfer of cage Me-**1** from the mixture*

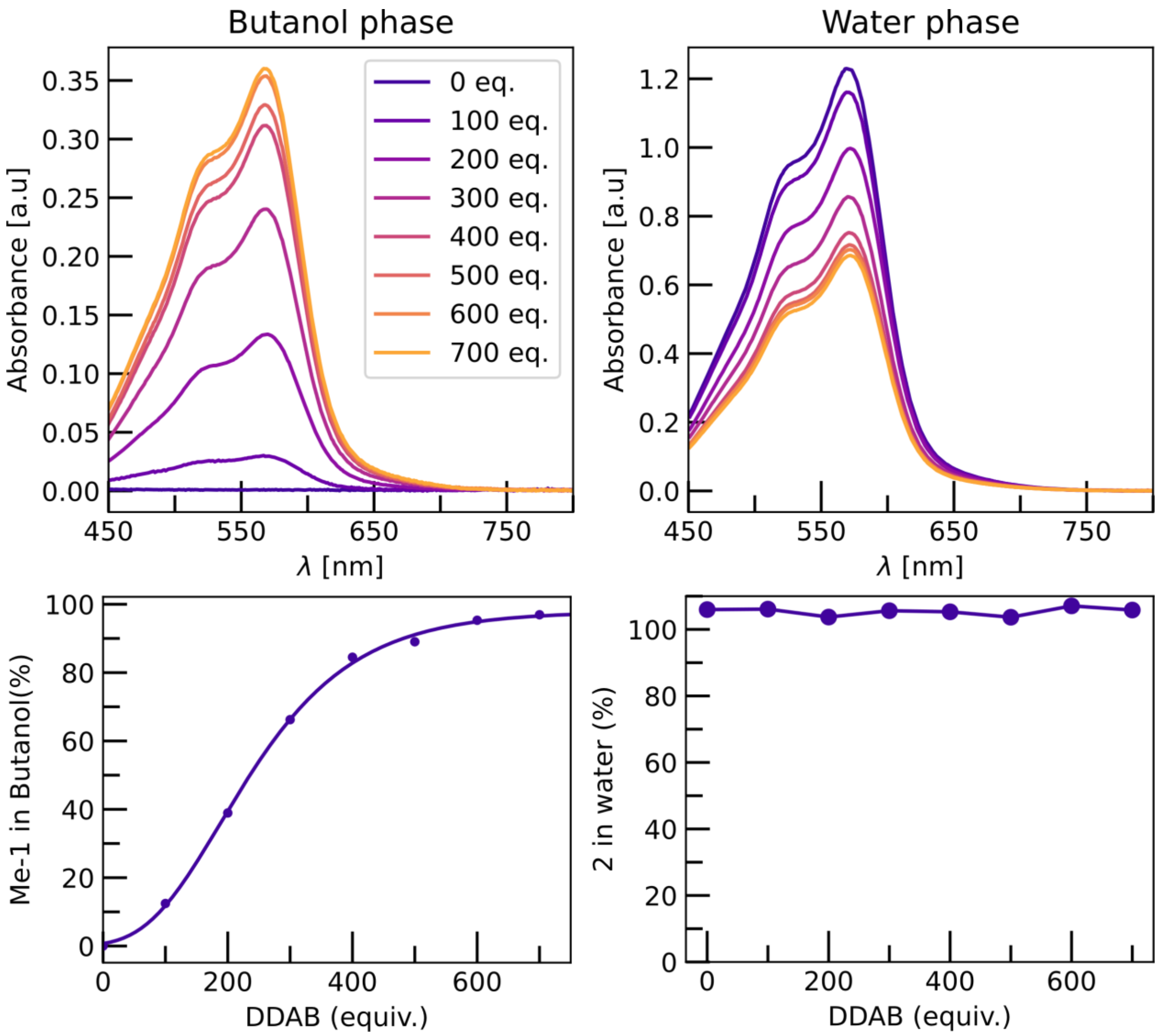

Figure S32. Left: UV-Vis absorbance spectra and phase transfer plot (1-butanol layer) for selective transfer of cage Me-**1** from the mixture. Right: UV-Vis spectra and proportion of cage **2** (water layer).

Table S26. Constants for fitted Gompertz curve in Figure S31.

Layer	*d*	*a*	*c*	*b*	R^2
1-Butanol	0	97.9	188.5	0.00846	0.999

Table S27. Summary of UV-Vis data.

Layer	DDA^+ (equiv.)	Abs	Cage Me-1 (μM)	Cage 2 (μM)
Water (λ = 571)	0	1.230	25.0	26.5
	100	1.160	22.1	26.5
	200	0.997	15.3	25.9
	300	0.857	9.39	26.4
	400	0.752	5.03	26.3
	500	0.717	3.54	25.9
	600	0.703	2.97	26.8
	700	0.686	2.26	26.5
1-Butanol (λ = 568)	0	0.002	0.00	-
	100	0.030	3.11	-
	200	0.134	9.73	-
	300	0.240	16.6	-
	400	0.312	21.1	-
	500	0.329	22.3	-
	600	0.354	23.8	-
	700	0.360	24.2	-

*S9.2.3 Selective transfer of cage **2** from the mixture*

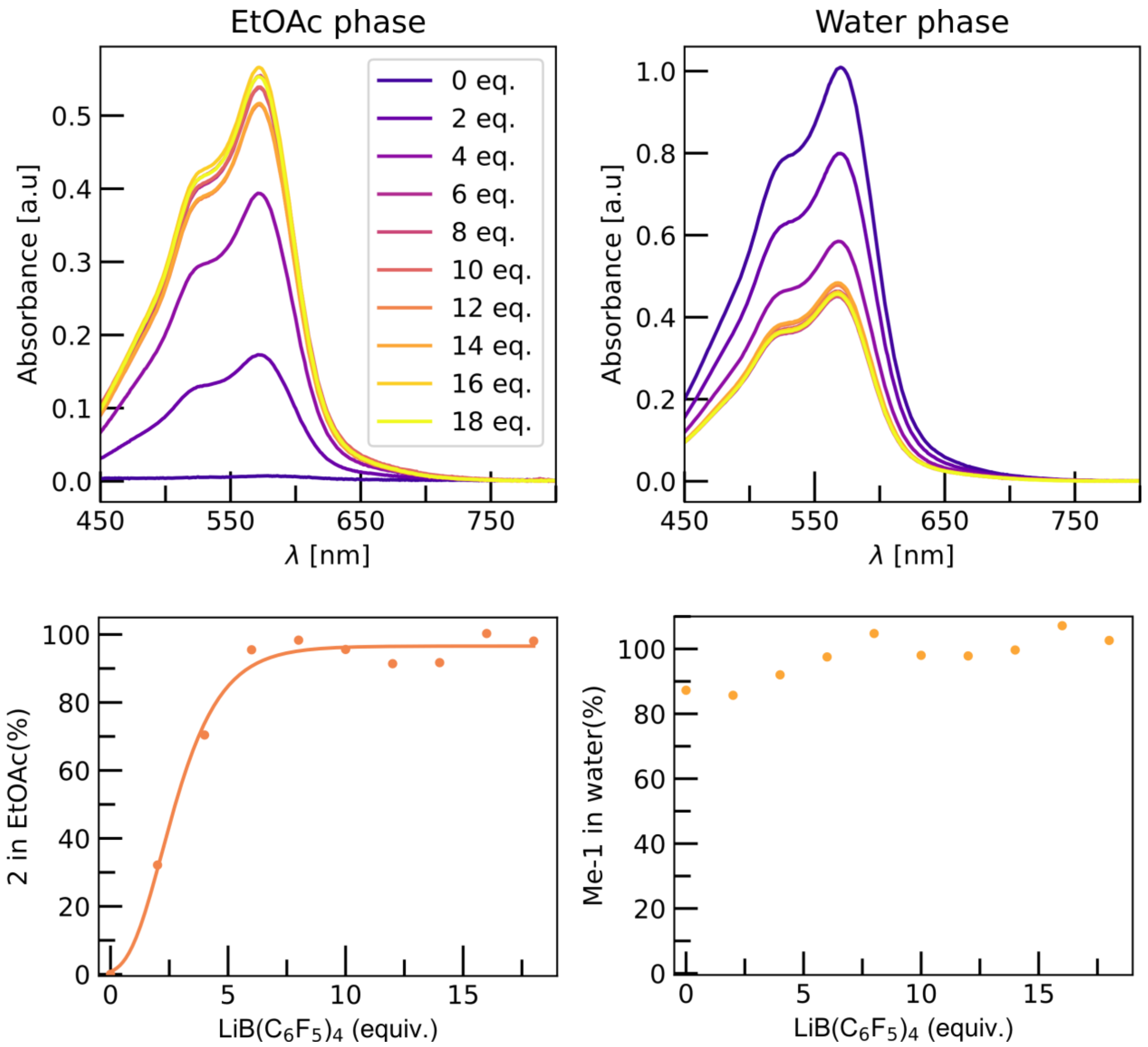

Figure S33. Left: UV-Vis absorbance spectra and phase transfer plot (ethyl acetate layer) for selective transfer of cage **2** from the mixture. Right: Proportion of cage Me-**1** in water.

Table S28. Constants for fitted Gompertz curves in Figure S32.

**Layer**	*d*	*a*	*c*	*b*	R^2
EtOAc	0	96.6	2.18	0.722	0.989

Table S29. Summary of UV-Vis data.

Layer	$[B(C_6F_5)_4]^-$ (equiv.)	Abs	Cage Me-1 (μM)	Cage 2 (μM)
Water (λ = 568)	0	1.009	21.8	25.0
	2	0.799	21.4	13.3
	4	0.585	23.0	1.37
	6	0.451	24.4	-6.13
	8	0.464	26.2	-5.39
	10	0.452	24.5	-6.04
	12	0.478	24.5	-4.61
	14	0.484	24.9	-4.28
	16	0.462	26.8	-5.49
	18	0.456	25.7	-5.81
Ethyl acetate (λ = 572)	0	0.007	-	0.00
	2	0.173	-	8.04
	4	0.394	-	17.6
	6	0.538	-	23.9
	8	0.555	-	24.6
	10	0.539	-	23.9
	12	0.515	-	22.9
	14	0.517	-	22.9
	16	0.566	-	25.1
	18	0.553	-	24.5

S9.3 Orthogonal phase transfer followed by NMR spectroscopy

A solution containing cage Me-**1** (~1 mM) and cage **2** (~1 mM) was prepared in D_2O. Aliquots this solution (1 mL each) were then transferred to two 5 mL Eppendorf tubes. To one of these tubes a minimum amount of $LiB(C_6F_5)_4$ was added as a stock solution in ethyl acetate (0.5 M), and the total volume was corrected to 2 mL. To the other tube a DDABr solution in 1-butanol (0.5 mM, 1 mL) was added. The tubes were then inverted carefully 10 times to allow for contact between the layers, while avoiding emulsion formation. The solutions were allowed to settle for approximately 10 minutes, and then samples were taken from each layer for analysis by ^{1}H NMR. The samples in organic solvent were measured first to avoid decomposition. The spectrometer was locked on the deuterium signal from a coaxial capillary containing D_2O for the samples from the solvent layers.

Baseline distortions due to the very large solvent signals were observed for the samples in 1-butanol and ethyl acetate. To minimize this effect, the amount of organic solvent in the sample was reduced by replacing some of the sample with a small immiscible D_2O layer. This reduces the base line distortions, but the solvent signals are still very large compared to the cage peaks. We therefore only show the aromatic region in the spectra.

Figure S34 (next page) shows the ^{1}H NMR spectra in the water and butanol layers after transfer of cage Me-**1** from the mixture. After transfer, only signals corresponding to cage **2** can be observed in the water layer. There is a small set of signals corresponding to free amine, these were also present in the initial solution before phase transfer, and originate from the solution of cage **2** used for this experiment. The peaks in the butanol layer belong exclusively to cage Me-**1** (compare with Figure S.30). No new subcomponent peaks can be observed in either layer, indicating no breakdown of the cages following phase transfer.

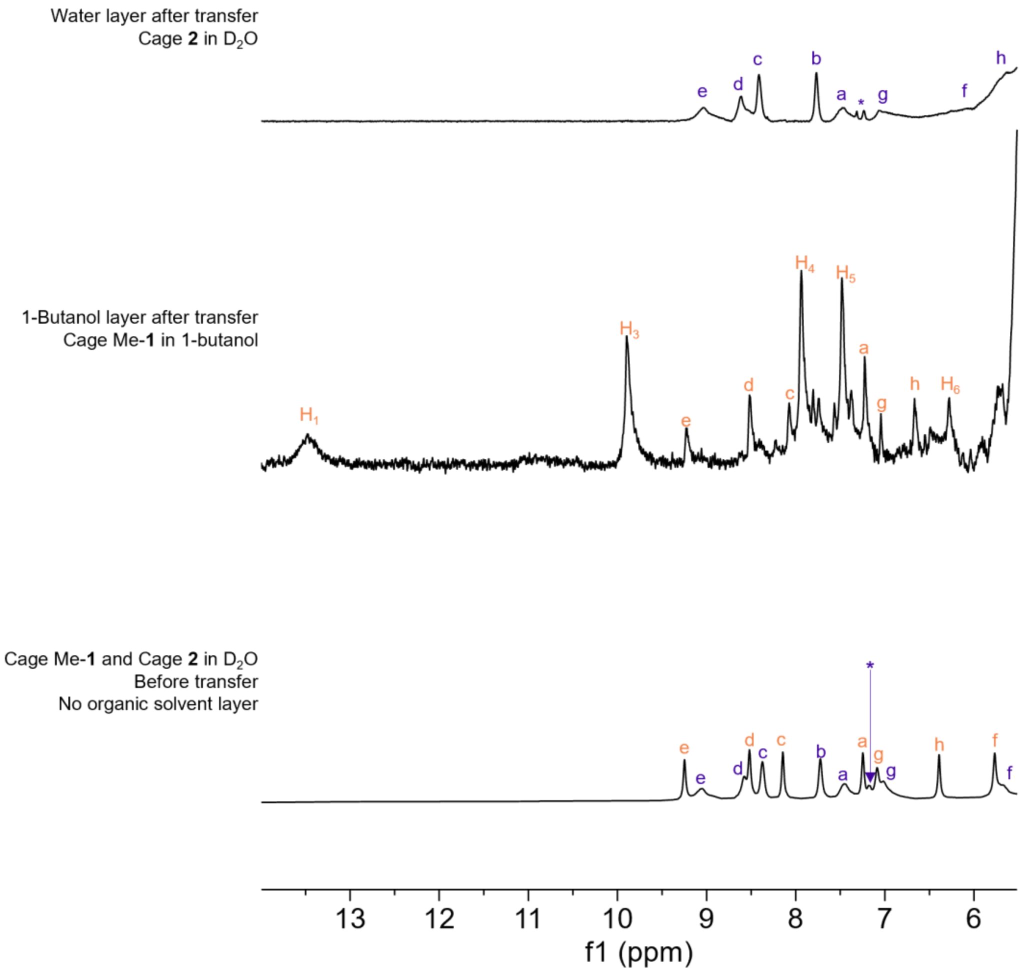

Figure S34. Stacked ^{1}H NMR spectra (800 MHz, 298 K, referenced to DSS) spectra of the transfer of cage Me-**1** from the mixture. Purple labels correspond to cage **2**, while orange labels correspond to cage Me-**1**. Purple stars indicate excess amine from initial solution of cage **2**.

Figure S35 (next page) shows the ^{1}H NMR spectra in water and ethyl acetate after transfer of cage **2** from the mixture. The ethyl acetate layer only contains peaks corresponding to cage **2** in ethyl acetate (see Figure S.21). The water layer after transfer contains two sets of signals, similar to the two sets of signals generated by cage Me-**1** in the presence of an immiscible 1-butanol layer. Neither set of signals were consistent with residual cage **2**. We therefore suspected that both sets of signals are attributable to cage Me-**1**. To confirm this, a solution only containing cage Me-**1** in D_2O was combined with ethyl acetate. The resulting ^{1}H NMR spectrum in D_2O contains two sets of signals from cage Me-**1**, and matches the water layer following transfer of cage **2** into ethyl acetate. No new subcomponent peaks can be observed in either layer, indicating no breakdown of the cages following phase transfer.

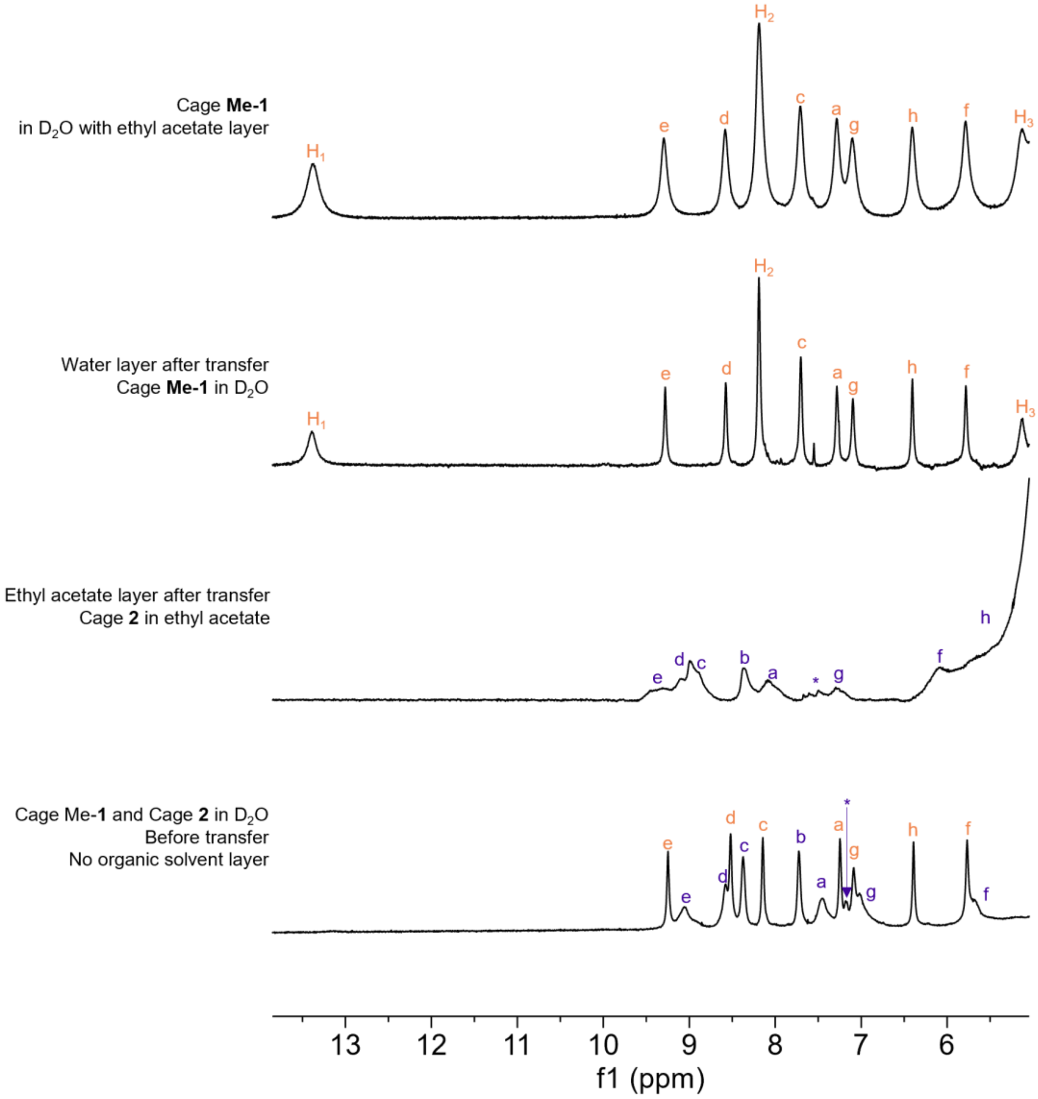

Figure S35. Stacked ^{1}H NMR spectra (800 MHz, 298 K, referenced to DSS) spectra of the transfer of cage **2** from the mixture. Purple labels correspond to cage **2**, while orange labels correspond to cage Me-**1**. Purple stars indicate excess amine from initial solution of cage **2**.

S10 Supporting references

[29] P. Mal, D. Schultz, K. Beyeh, K. Rissanen, J. R. Nitschke, *Angew. Chem. Int. Ed.* **2008**, *47*, 8297–8301.

[30] O. V Dolomanov, L. J. Bourhis, R. J. Gildea, J. A. K. Howard, H. Puschmann, *J. Appl. Crystallogr.* **2009**, *42*, 339–341.

[31] G. M. Sheldrick, *Acta Crystallogr. Sect. C* **2015**, *71*, 3–8.

[32] I. A. Guzei, *J. Appl. Crystallogr.* **2014**, *47*, 806–809.

[33] P. van der Sluis, A. L. Spek, *Acta Crystallogr. Sect. A* **1990**, *46*, 194–201.

[34] J. B. Maglic, R. Lavendomme, *J. Appl. Crystallogr.* **2022**, *55*, 1033–1044.
